# Study design and the sampling of deleterious rare variants in biobank-scale datasets

**DOI:** 10.1073/pnas.2425196122

**Published:** 2025-06-03

**Authors:** Margaret C. Steiner, Daniel P. Rice, Arjun Biddanda, Mariadaria K. Ianni-Ravn, Christian Porras, John Novembre

**Affiliations:** ^a^Department of Human Genetics, University of Chicago, Chicago, IL 60637; ^b^Media Laboratory, Massachusetts Institute of Technology, Cambridge, MA 02139; ^c^SecureBio, Cambridge, MA 02142; ^d^Department of Biology, Johns Hopkins University, Baltimore, MD 21218; ^e^Department of Genetics and Genomic Sciences, Icahn School of Medicine, New York, NY 10029; ^f^Department of Ecology and Evolution, University of Chicago, Chicago, IL 60637

**Keywords:** population genetics, rare variants, negative selection

## Abstract

One key component of study design in population genetics is the “geographic breadth” of a sample (i.e., how broad a region across which individuals are sampled). How the geographic breadth of a sample impacts observations of rare, deleterious variants is unclear, even though such variants are of particular interest for biomedical and evolutionary applications. Here, in order to gain insight into the effects of sample design on ascertained genetic variants, we formulate a stochastic model of dispersal, genetic drift, selection, mutation, and geographically concentrated sampling. We use this model to understand the effects of the geographic breadth of sampling effort on the discovery of negatively selected variants. We find that samples which are more geographically broad will discover a greater number of variants as compared to geographically narrow samples (an effect we label “discovery”); though the variants will be detected at lower average frequency than in narrow samples (e.g., as singletons, an effect we label “dilution”). Importantly, these effects are amplified for larger sample sizes and fitness effects. We validate these results using both population genetic simulations and empirical analyses in the UK Biobank. Our results are particularly important in two contexts: the association of large-effect rare variants with particular phenotypes and the inference of negative selection from allele frequency data. Overall, our findings emphasize the importance of considering geographic breadth when designing and carrying out genetic studies, especially at biobank scale.

## Significance

As genetic studies grow, researchers are increasingly seeking to identify rare genetic variants with large impacts on traits. In this paper, we combine theoretical methods and data analysis to show how differences in sampling with respect to geographic location can influence the number and frequency of genetic variants that are found. Our results suggest that geographically broad samples will include more distinct genetic variants, though each variant will be found at a lower frequency, as compared to geographically narrow samples. Our results can help researchers to consider the implications of study design on expected results when constructing new genetic samples.

In recent decades, the size of genetic sequencing cohorts has grown exponentially. Nowhere is this more evident than in human genetics, where the launch of biobanks has transformed the paradigm of data analysis such that sample sizes in the hundreds of thousands are increasingly commonplace (1). Yet, the largest and most commonly utilized biobank-scale genomics datasets are heavily biased toward individuals of European ancestries (2–5), leading to scientific and ethical concerns for precision medicine (6, 7). As a response to this, new biobanks have been launched with specific purposes to diversify available genomics data (8–11). Consequently, not only is the size of human genetics data continuing to increase but the geographic and genetic spaces from which individuals are sampled are growing dramatically.

This development in the field has clear benefits for improving representation in human genetics research and the transferability of results across diverse populations (12–14). What has yet to be addressed is how this change in study design will affect the results of genetic studies at the level of discovered variants. Motivated as such, we ask, as the geographic breadth of a genetic study increases, how should one expect the number and frequency of discovered variants to change? That is, how is the site frequency spectrum (SFS) of observed variants affected by the geographic breadth of a sample? The answer to this question has significant implications for studies in human genetics and genetics more broadly.

For understanding the genetic basis of traits, this question is of interest, as sample design likely impacts the discovery of genetic associations to phenotypes. A key focus of biomedical applications is discovering variants that have large effects on disease susceptibility, as such variants may provide the most biological insight into the etiology of disease and in turn potential therapeutic paths (15, 16). From evolutionary principles, one expects large effect genetic changes most often to be kept at very low population frequencies by the action of natural selection (either due to simple negative selection or via underdominance induced by stabilizing selection; 17). Indeed, rare, deleterious variants have been shown to be enriched in genomic regions of functional interest such as drug target regions (18), have yielded numerous associations with phenotypic outcomes (19, 20), and are argued to be a key component of unexplained heritability in human traits (21). How the geographic breadth of sampling impacts the discovery of these rare, deleterious variants is unknown, yet crucial to the design of studies which aim to characterize such variants.

Understanding how sample design affects the observed SFS of deleterious variants is also important to evolutionary geneticists. In evolutionary genetics, a persistent goal has been to characterize the distribution of fitness effects (DFE)—i.e., the probability with which newly arising mutations are deleterious, advantageous, or selectively neutral—using allele frequency data (22–25), in part because of its implications for genome evolution, mutational load, and conservation efforts (26). Inference of the DFE from population genetic data depends on measurements of the number and frequencies of deleterious variants (equivalently, the SFS). In addition, the SFS in large samples can be used to estimate mutation and demographic parameters (e.g., refs. 27 and 28). Thus, understanding whether and how the geographic breadth of sampling impacts the observed SFS of deleterious variants is also important in order to avoid potential biases in SFS-based inferences of evolutionary parameters.

While the geographic distribution of deleterious variants has relevance in the context of contemporary biobank-scale human and evolutionary genetics, versions of the same question have been explored in the history of population genetics. The earliest interest in this topic was framed in terms of understanding the allele frequencies of recessive lethal variants and the closely related notion of understanding rates of “allelism” of lethal variants (i.e., the rate at which carriers of distinct recessive lethal alleles fail to survive). Notably, Wright and Dobzhansky studied rates of allelism in lethal variants from spatially separated Drosophila populations as early as the 1940s (29, 30). Bruce Wallace later carried out a notable survey to characterize how the rate of allelism of lethals decays as a function of geographic distance (31) and found that the allelism of lethal mutations decayed exponentially with the square root of distance in Drosophila melanogaster samples. In a parallel line of theoretical work developing the stepping-stone model of migration, Weiss and Kimura derived an expression for the spatial covariance of allele frequencies under neutrality in two dimensions (32). Maruyama later extended this to incorporate the effects of negative selection and considered continuous spatial models (33). Motivated by the work of Wallace (31), Yokoyama modeled the rate of allelism of lethals and the closely related covariance in allele frequency as a function of distance in the stepping-stone model (34).

In a second related line of research, motivated by understanding the consequences of spatial structure and sampling on the inference of demographic history, several previous studies have investigated the effect of sample design on neutral variation (35–43). These studies emphasize how in most cases, geographically concentrated (or “narrow”) sampling in spatial populations leads to a shift in the neutral SFS with a decrease in observed singletons and enrichment of intermediate and high frequency alleles (i.e. negative Tajima’s D; 44).

Thus, the spatial distribution of carriers of deleterious alleles has been a topic of interest in population genetics for over eighty years. Notably, though, previous studies have not considered sample sizes on the scale of modern human biobank cohorts, which reach tens to hundreds of thousands of individuals, nor have they addressed the extent to which distortions of the SFS are amplified or diminished for rare, deleterious variants.

Here, with a focus on the discovery of rare, deleterious variants, we develop a theoretical analysis to obtain the expected counts in the sample SFS from a spatially structured population that is sampled nonuniformly. To do so, we utilize recent mathematical results on the stationary distribution of subcritical measure-valued branching processes with immigration by Friesen et al. (45). The model considers the distribution of carriers of deleterious alleles in continuous geographic space—accounting for dispersal, genetic drift, selection, mutation, and sampling simultaneously—and we derive results that allow the rapid computation of the expected sample SFS across a large range of parameter values

As important background, we note that in the panmictic (or “well-mixed”) case, allele frequencies for deleterious variants are well known to follow a two-parameter distribution, such that the probability density g(x) for an allele under negative selection appears at frequency *x* follows (46, 47):

[1] $$\begin{array}{c} g\left(x\right)\propto e^{-\gamma x}{\left[x\left(1-x\right)\right]}^{\theta -1}, \end{array}$$

where *γ* is the population-scaled selection coefficient ($\gamma =4N_{e}s$ with *N*_*e*_ being the effective population size and *s* is the strength of negative selection acting on heterozygotes, *s* ≥ 0) and *θ* is the population-scaled mutation rate ($\theta =4N_{e}\mu$ with mutation rate *μ* per site per generation). For variants under negative selection (*γ* > 0), the exponential term ($e^{-\gamma x}$) induces a reduction in the abundance of observed alleles as a function of the allele frequency *x*. This mirrors the intuition that alleles under negative selection are less likely to reach higher frequencies.

As we will show, when considering spatially restricted dispersal and geographically concentrated sampling, allele frequencies still follow a two-parameter distribution with scaled “effective” selection (*γ*_*E*_) and mutation (*θ*_*E*_) terms. However, these terms are now dependent on the spatial scale of the sampling effort and the offspring dispersal scale, in addition to the usual mutation, selection, and population size parameters. We subscript the parameters with “E” to emphasize that the effects of spatial sampling can be obtained by using these effective parameters in the standard formulas for the SFS of negatively selected alleles. The resulting distributions show that the geographic breadth of a sample has strong effects on the SFS as well as downstream summary statistics, and we assess how these effects change with increasing sample size and selection strength.

We validate our theoretical results using simulations that share our modeling assumptions as well as in a more realistic, individual-based spatial model (42, 48). As continuous-space simulations can be computationally intensive, our development of theoretical approximations allows us to efficiently gain insights across wide parameter ranges without using simulation.

To address the effects of geographically concentrated sampling empirically, we also conduct in silico resampling experiments using the UK Biobank exome sequencing dataset (19) to measure the impact of sampling at different geographic scales. The results broadly confirm our theoretical predictions and yield insights into how sampling design impacts the number of human genetic variants discovered and their frequencies.

## Methods

### Population Genetic Model.

**Fig. 1.**
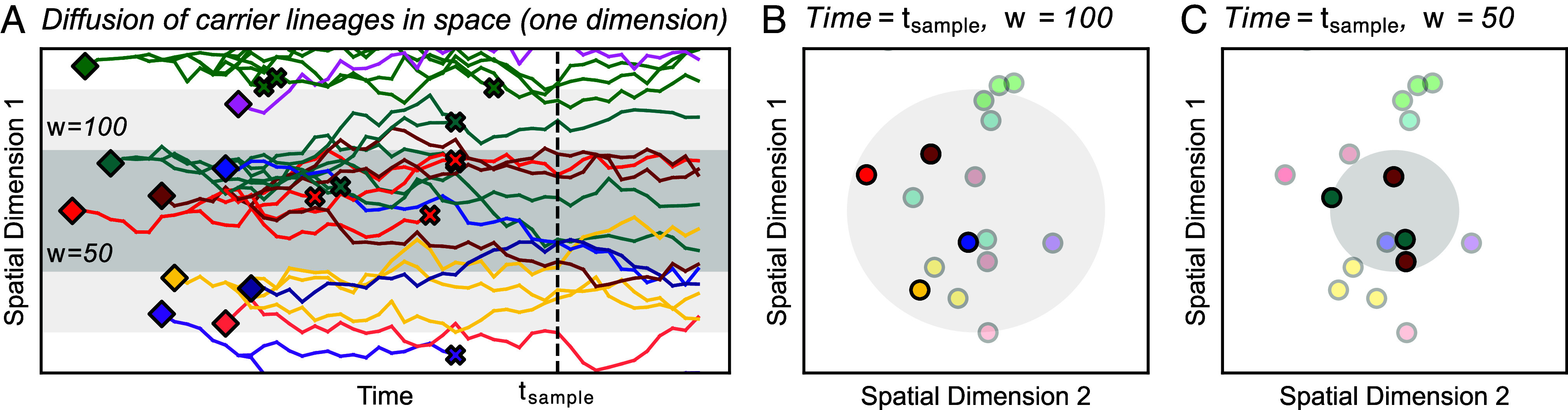
Illustration of a spatial branching process model with sampling. (*A*) As time progresses, carrier lineages move in space (diffusion), branch into sublineages (reproduction), and die. Diamonds and X’s denote lineage birth and death, respectively. For simplicity, we show only one spatial dimension on the vertical axis. Shaded areas represent sampled areas with widths *w* = 50 and *w* = 100. (*B* and *C*) Here, we visualize the locations of carriers from panel (*A*) at a particular time indicated as *t*_*sample*_. Within the sampled area, rare variant carriers can be discovered and included in the sample (opaque points). In this example, sampling from the broader area (*w* = 100) results in a greater number of distinct mutations being observed, all as singletons. The narrow sample (*w* = 50) discovers two distinct mutations with each as a pair of doubletons. This toy example illustrates the potential effects of sampling breadth on entries of the sample SFS (here, the counts of singletons vs. doubletons).

We model how each instance of a rare allele is born, moves in space, reproduces, and dies in a two-dimensional continuous geographic habitat of size *L* × *L*. In our model (Fig. 1), deleterious alleles are generated by de novo mutation according to a Poisson point process with rate $\rho_{N}\mu$, where *ρ*_*N*_ is the population density and *μ* is the per-generation mutation rate. We note that, in our model, *ρ*_*N*_ and *L* are constants, which implies an assumption of constant population size. Each de novo allele appears at a random location in the habitat and migrates according to a homogeneous, isotropic diffusion process. In other words, the dispersal process is the same across the habitat and movement is uniform in all directions. With diffusion coefficient *σ*^2^ and time measured in generations, the per-generation displacement in two-dimensional space follows a bivariate Gaussian distribution with SD *σ* in each dimension (with zero covariance). Following other classical analyses of spatial models (32, 49, 50), we assume for simplicity that the habitat has periodic boundary conditions (i.e., the habitat is a two-dimensional torus, and so there will be no boundary effects).

Within the habitat, we model reproduction and death as a continuous time branching process, a type of stochastic process which has frequently been applied in theoretical population genetics for rare variants (see, for instance, refs. 51–54). During their lifetime, each instance of a deleterious allele gives rise to offspring alleles with rate 1−s and dies at a rate 1. A larger positive value of *s* indicates stronger negative selection. We note that our term *s* is equivalent to the *hs* parameter used in alternative parameterizations of selection where *h* is a general dominance parameter and *s* is a selection coefficient describing the fitness of homozygous carriers (e.g., ref. 55). We assume deleterious variants are rare enough that the fraction of homozygous carriers is negligible and alleles can be approximated in a haploid model with alleles evolving independently. Also, while we model negative selection on individual variants, these dynamics are similar to those of newly arising variants which affect complex traits under stabilizing selection (which experience a form of underdominance, see ref. 17). The use of a branching process model implies that each allele copy evolves independently of one another and of the ancestral alleles (similar to refs. 56–58). In the context of continuous space, the assumption of independence of the branching process and dispersal process are approximate (e.g., see ref. 50), but hold reasonably when every mutation is locally rare.

### Modeling Geographically Concentrated Sampling.

The spatial model in the previous section describes the process by which rare variants arise and disperse in geographic space. Our next step is to define how sampling of this spatial population occurs. To this end, we model the probability that an individual at a particular position within the habitat is included in the sample. We posit a sampling “center” and assume that the probability of an individual being sampled is determined by the individual’s distance from that center using a particular distribution (the “sampling kernel;” *SI Appendix*, Fig. S1). The SD of the sampling kernel, which we denote by *w*, determines the breadth of sampling effort—or “sampling breadth”—i.e., the extent to which sampling effort is distributed across the habitat. On one extreme, for *w* ≳ *L*, the sampling process converges to “uniform” sampling in which all individuals have an equal probability of being sampled, regardless of spatial position. In the other extreme, as *w* becomes small, the sampling kernel approaches “point sampling” in which all sampled individuals are located at the same position. In between these two limiting cases, the value of *w* will determine how spatially “broad” (larger *w*) or “narrow” (smaller *w*) a sample will be.

In our implementation, the sampling kernel has the form of a Gaussian distribution with SD *w*, though we note that our methods are generalizable to other sampling kernels. We employ the Gaussian sampling model to approximate the sampling processes used in constructing real genetic samples, such as sampling centered at field stations for ecological genetics or in biomedical centers for human genetics. As in our population genetic model, the sampling model has periodic boundary conditions (i.e., there are no “edge effects” in our model). This construction is appropriate when the habitat size, *L*, is sufficiently large compared to the sampling breadth, *w*, such that we can ignore behavior as the sampling kernel hits the edge of the habitat. Our simulations will show that in cases where *w* approaches the scale of *L*, the results converge to those of uniform random sampling.

### Theoretical Analysis Methods.

In order to solve for the SFS, we frame the problem as special case of a spatial subcritical measure-valued branching process (see refs. 54, 59, and 60). Applying results of Friesen et al. (45), we obtain a nonlinear partial differential equation (PDE) governing the spatially weighted population allele frequency distribution at stationarity. We solve this PDE perturbatively to obtain the mean and variance of the spatially weighted allele frequency. Based on the uniform sampling case and exploratory simulations, we inferred that the full stationary distribution approximately follows a Gamma distribution, and we find the parameters of the Gamma distribution by matching moments.

Having derived the stationary distribution of spatially weighted population allele frequencies, it remains to obtain the SFS for a sample of finite size. For a large sample size *n*, the number of sampled rare alleles conditional on the population allele frequency approximately follows a Poisson distribution. Then, by a property of Gamma-Poisson mixture distributions, the per-site SFS (i.e. the probability a given site takes on an observed count of 0,1,…,n) follows a Negative Binomial distribution with parameters determined by the shape and rate of the Gamma distribution as well as the sample size *n*. Last, we use the finite-sample SFS results to derive expectations of several major population genetic summary statistics.

We refer readers to the extended theoretical methods in *SI Appendix* for a detailed description of these methods.

### Population Genetic Simulations.

We validate our theoretical results with two sets of simulations. First, we simulate a spatial branching process in a two-dimensional continuous habitat and sample according to a Gaussian sampling density, as our theory assumes. These simulations are close to our theory in that they make the same rare-allele approximation. Their role is to check the analytical approximations we make in the course of deriving the SFS.

In addition to the branching process simulations, which align directly to our theoretical model, we carried out more realistic forward-time, individual-based population genetic simulations in SLiM (48) using identical conditions to ref. 42 except that all variants are deleterious with some selection coefficient. In contrast to our other simulations, the SLiM model contains multiple stages of the life cycle, models diploid genomes, and—crucially—does not assume variants are rare and independently evolving.

We refer the reader to *SI Appendix* for additional details on simulation methods. All simulation code and associated scripts are available at: https://doi.org/10.5281/zenodo.15398319 (61).

### Analysis of Whole Exome Sequencing Data from the UK Biobank.

We perform resampling experiments using the whole exome sequencing dataset (n=469,835) in the UK Biobank (UKB; 19) in order to assess the predicted effects of sampling breadth on sample allele frequencies and associated summary statistics. After including only individuals which met quality control and relatedness thresholds used by Bycroft et al. (4), we subset the data to individuals both born within the United Kingdom and having similar genetic ancestry (specifically, individuals within 0.0001 units in Euclidean distance from the centroid in the normalized PC1-PC2 space; applying both filters results in n=231,073 individuals). We then use a sampling importance resampling (SIR) method to construct n=10,000 samples such that the distribution of birthplace coordinate bins is Gaussian with centers centered at each of three geographic points with SD (*w*) set to 50 km, 100 km, and 150 km, as well as a uniform sample (see *SI Appendix* for details on the sampling algorithm). We repeat the sampling procedure ten times for each sampling width and center.

For each weighted subsample, we compute the SFS for putative LoF sites on chromosome 1 (32,320 variants) as well as equal-sized random subsets of synonymous and missense variants (subsets generated using PLINK v1.90b6.26; variant annotation provided by UKB). We then calculate summary statistics (number of variant sites, number of singletons, and heterozygosity). We refer the reader to the *SI Appendix* for additional details.

## Results

### The Finite Sample SFS Depends on Ratios Between Spatial Scales as Well as Sample Size.

In our model, a key emergent feature is the length scale across which an allele spreads during the time from the initial mutation to the extinction of all its descendant copies, which we denote as *ℓ*_*c*_, the characteristic length scale for this problem. As the lineages diffuse with coefficient *σ*^2^ and alleles go extinct on a time-scale of 1/s generations, $\ell_{c}=\sqrt{\sigma^{2}/s}$. We note that the same ratio arises in related but different models in the spatial population genetics literature (for instance, refs. 62, 63). In our model, alleles that spread more quickly (large *σ*) will move farther during the lifespan of the allele (*ℓ*_*c*_ is large). Conversely, alleles which are under stronger negative selection (large *s*) die more quickly and thus the descendant alleles will have spread over shorter lengths (*ℓ*_*c*_ is small). As we will see later in our results, how the spatial scale of the sampling effort (*w*) compares to the spatial spread of the allele (*ℓ*_*c*_) will be an important factor in the behavior of the SFS.

In order to derive the form of the SFS for a finite sample of size *n* with sampling breadth *w*, we first consider the distribution of allele frequencies across the entire spatially extended population with a weighting on each position provided by the sampling kernel. In a panmictic population, the population SFS of rare, deleterious alleles approximately follows a Gamma density (by ignoring the x→1 tail of Eq. **1**). We show analytically that this approximation holds for spatially uniform samples under our model (*SI Appendix*) and confirm via simulations that allele frequencies of spatially concentrated samples are also well-approximated by a Gamma distribution (*SI Appendix*, Figs. S2 and S3). Intuitively, the Gamma distribution captures two important effects: power-law behavior at low frequencies due to mutation-drift balance and an exponentially decaying tail at high frequencies due to selection.

We analyze our model in order to obtain the two parameters of this Gamma distribution, which we refer to as the effective mutation supply, *θ*_*E*_, and the effective selection intensity, *γ*_*E*_. Then, we derive an expression for the expected SFS of a finite sample with size *n* in terms of these parameters. First, let the random variable *K* denote the count of derived alleles at a single site in a sample of size *n*. Given a Gamma density for the allele frequencies, and approximating the binomial sampling process for large *n* as a Poisson distribution, *K* follows a Negative Binomial distribution with number of successes *θ*_*E*_ and success probability $\gamma_{E}/\left(\gamma_{E}+n\right)$:

[2] $$\begin{array}{c} K∼\;\text{NegBin}\left(\theta_{E},\frac{\gamma_{E}}{\gamma_{E}+n}\right). \end{array}$$

The *k*-th entry of the normalized sample SFS is then given by $\xi_{k}^{\left(n\right)}\equiv \;\text{Pr}\left(K=k\right)$. In the limit that *θ*_*E*_ is small, this becomes approximately:

[3] $$\begin{array}{c} \xi_{k}^{\left(n\right)}=\frac{\theta_{E}}{k}{\left(\frac{n}{\gamma_{E}+n}\right)}^{k}. \end{array}$$

In the case of spatially concentrated sampling (*w* ≪ *L*), we show (*SI Appendix*) that the effective mutation and selection terms are given by

[4] $$\begin{array}{c} \theta_{E}\equiv \mu \rho_{N}\ell_{c}^{2}\lambda , \end{array}$$

and

[5] $$\begin{array}{c} \gamma_{E}\equiv s\rho_{N}\ell_{c}^{2}\lambda , \end{array}$$

where $\rho_{N}\ell_{c}^{2}$ is akin to a population size. We denote by *λ* a term we refer to as the sampling effect scalar, which is a function of $w/\ell_{c}$:

[6] $$\begin{array}{c} \lambda \equiv \frac{4\pi }{\;\text{exp}\left({\left(w/\ell_{c}\right)}^{2}\right)E_{1}\left({\left(w/\ell_{c}\right)}^{2}\right)}, \end{array}$$

where $E_{1}\left(x\right)=\int_{1}^{\infty }\left(e^{-tx}dt\right)/t$ is the exponential integral function.

We note that in Eq. **5**, the leading selection coefficient *s* cancels with the 1/s term in $\ell_{c}^{2}$, meaning that the only dependence of either effective parameter on selection is via *λ*.

**Fig. 2.**
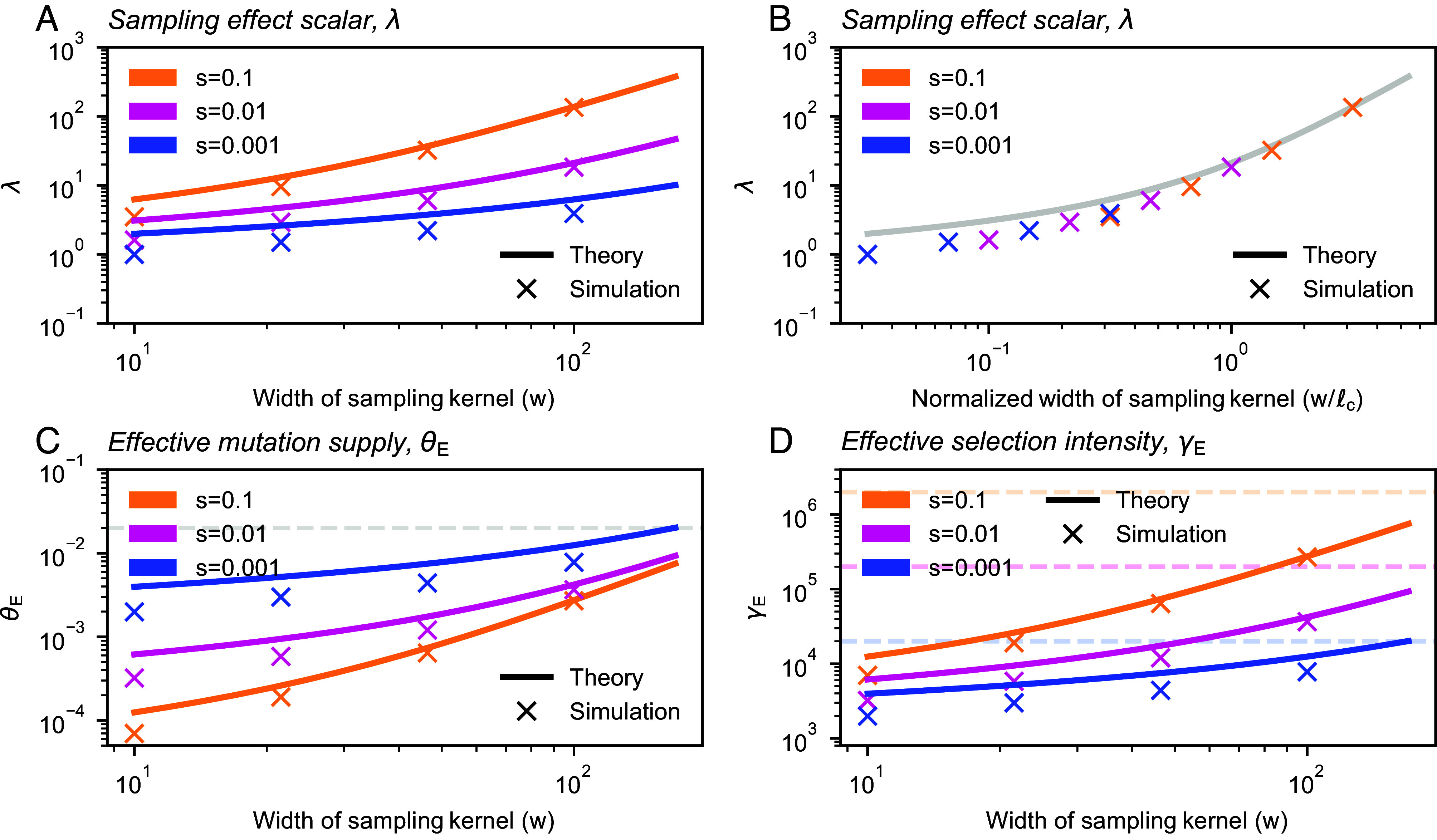
The sampling effect scalar and effective parameters of the SFS. (*A*) As the breadth of the sampling kernel (*w*) increases, the sampling effect scalar *λ* also increases. (*B*) When plotted as a function of $w/\ell_{c}$, the relationship is identical across a range of selection intensities. (*C* and *D*) Both the effective mutation supply, *θ*_*E*_, and the effective selection intensity, *γ*_*E*_, depend on the selection coefficient (via *ℓ*_*c*_) and the breadth of the sampling kernel. Dashed lines show values of *θ*_*E*_ and *γ*_*E*_ for the uniform case in panels (*C* and *D*), respectively. Other parameters are *σ* = 10, *ρ* = 20, and $\mu =10^{-9}$. Branching process simulations shown are from the Gillespie algorithm run with a habitat size of L=1,000.

The value of *λ* increases as *w* increases (Fig. 2*A*) and depends on the ratio between the sampling breadth and characteristic length scale: $w/\ell_{c}$ (Fig. 2*B*). When $w/\ell_{c}$ is small (≪1), *λ* is approximately $-2\pi /\;\text{ln}(w/\ell_{c})$ and thus is only logarithmically dependent on *w* and *s* in this regime. Conversely, as $w/\ell_{c}$ becomes large, *λ* becomes approximately $4\pi {\left(w/\ell_{c}\right)}^{2}$ (Fig. 2*B*), in which case the effective parameters simplify to $\theta_{E}=\mu \rho_{N}4\pi w^{2}$ and $\gamma_{E}=s\rho_{N}4\pi w^{2}$ (Fig. 2 *C* and *D*). In this regime, the effective parameters scale quadratically with *w*, and are the equivalent to what one would expect for a panmictic population of size $\rho_{N}\pi {\left(2w\right)}^{2}$, which is interpretable as the size of a population of density *ρ*_*N*_ found in a circle whose radius is given by two times the sampling breadth (2w).

As *w* approaches *L*, both effective parameters eventually converge to their values in the uniform sampling limit (Nμ and *Ns*, respectively, where *N* is the total population size, similarly to Eq. **1**; Fig. 2 *C* and *D*). To understand this result, one can think of the term $\rho_{N}\ell_{c}^{2}\lambda$ as approximating the size of the population effectively being sampled, which grows as $\rho_{N}\pi {\left(2w\right)}^{2}$ before converging to $\rho_{N}L^{2}=N$ as sampling approaches the uniform case.

### Selection and Sampling Induce a Trade-Off Between Discovery and Dilution.

**Fig. 3.**
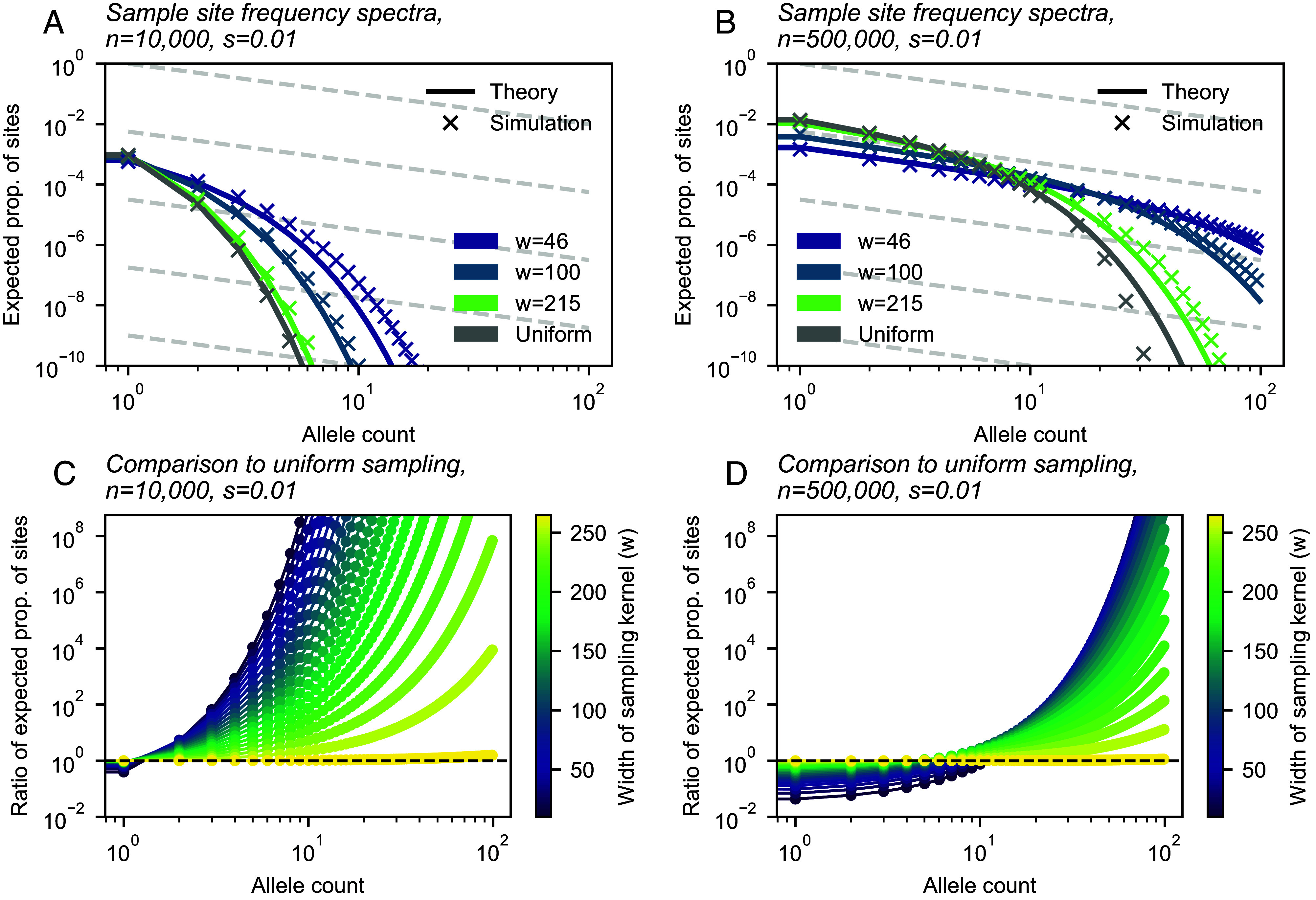
Site frequency spectra for a range of sampling widths. (*A* and *B*) Sample site frequency spectra for n=10,000 and n=500,000 sample sizes, respectively, shown for three sampling breadths (wrapped Gaussian sampling) and uniform sampling. (*C* and *D*) Ratio between frequency spectra elements for a range of *w* values (Gaussian sampling), relative to those of the SFS under uniform sampling, for parameter regimes shown in (*A* and *B*). For all panels, *σ* = 10, *ρ* = 20, $\mu =10^{-9}$, and *s* = 0.01. Branching process simulations shown were run with a habitat size of L=1,000.

Having derived an expression for the sample SFS, we now consider its behavior with respect to sampling breadth (Fig. 3 *A* and *B*). As expected from the effective parameters, we find that broader sampling effort (larger *w*) induces an upward shift in the intercept of the SFS on the vertical axis. We also observe a decrease in the frequency of segregating variants for broader samples. These results arise from what we call a “discovery” effect and a “dilution” effect. As the geographic breadth of a sample increases, the number of potential localized mutations one can find grows, and this is reflected in the increase in mutation supply (*θ*_*E*_) and the expected number of variants discovered (the discovery effect). At the same time, for a broader sample, each sampled deleterious variant is found at “diluted” frequencies because sampling broadly inadvertently captures many carriers of the alternative allele at each site (dilution effect). This is reflected in the larger *γ*_*E*_ term and concomitant faster rate of decay of the number of observed sites of count *k* as *k* increases. As sampling effort broadens (*w* increases), the SFS converges to the result under uniform sampling. Conversely, geographically narrow samples will capture fewer variants, but they are “concentrated” in the sample, meaning that they are observed at higher sampled allele frequencies on average than they would be found otherwise in a uniform sample.

Besides displaying the SFS directly, we visualize these results by comparing each entry of the SFS of a sample with breadth *w* to the SFS in the uniform limit (Fig. 3 *C* and *D*). The results show how relative to a uniform sample, as one makes the sample increasingly narrow in geographic breadth (*w* decreases), more deleterious alleles are found at higher allele counts and fewer are found at the lowest allele counts. At some value of allele count, the frequency spectra for a sample with breadth *w* and a uniform value are equal (which we see as the intersection between curves in Fig. 3 *A* and *B*), and the allele count at which the frequency spectra intersect is dependent on the sample size, *n*: For smaller samples, the intersection point is low, perhaps at the level of singletons; for larger samples, the intersection point is higher and the magnitude of effect in the low allele count range is also much larger (Fig. 3).

**Fig. 4.**
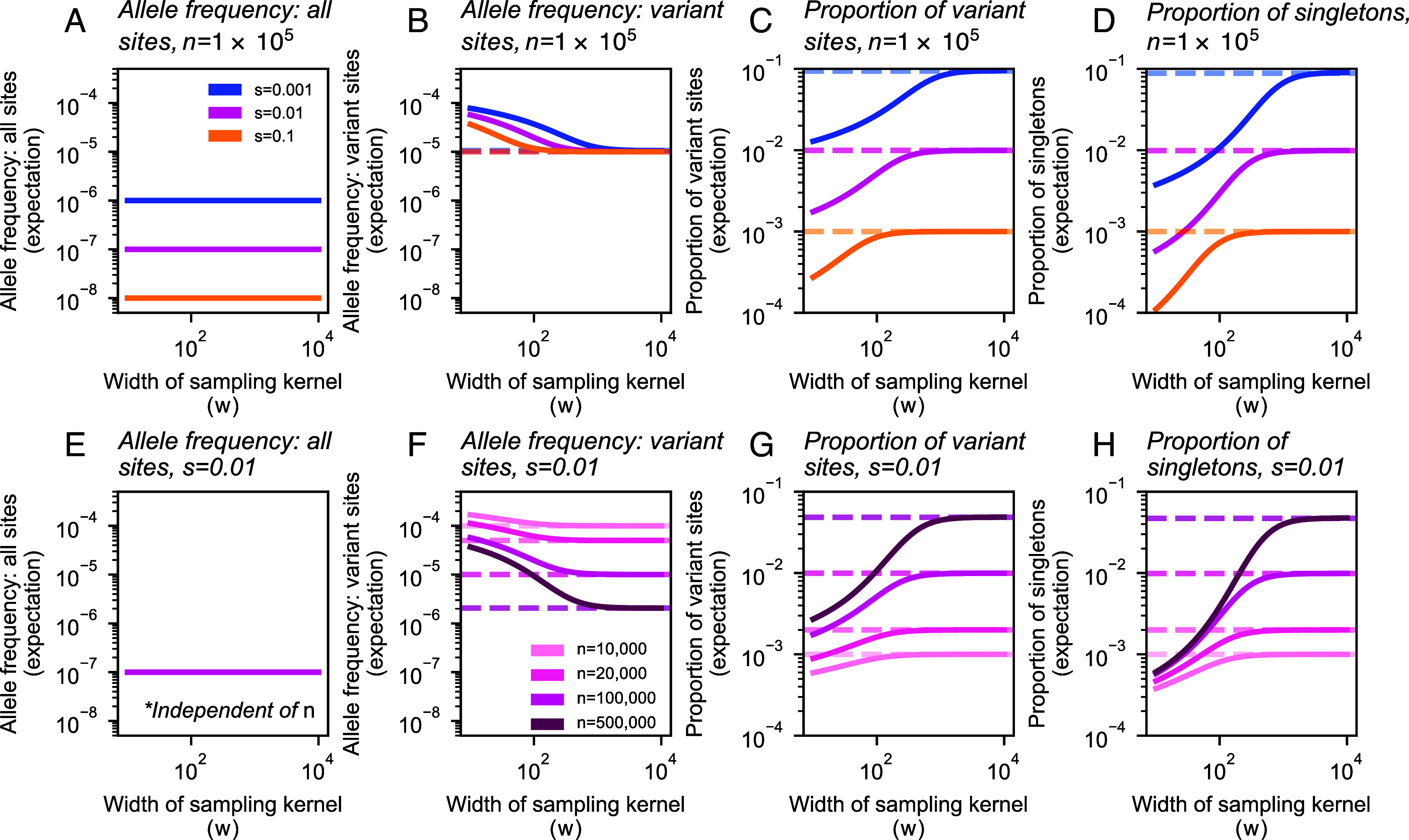
Expected values of summary statistics as a function of the width of the sampling kernel (*w*). (*A*–*D*) As the breadth of the sampling kernel increases, our model implies that the expected frequency across all sites will remain constant, though the expected frequency of variant sites will decrease. The expected proportion of variant sites and the expected proportion of singletons will both increase. Values for these statistics (excluding expected frequency across all sites, *A*) converge to the theoretical expectation under uniform sampling (dashed lines) as *w* increases, with convergence occurring more quickly for stronger selection coefficients. For more deleterious variants (larger *s*), the expected proportions of variant sites and singletons, as well as expected frequency across all sites, are shifted downward across the range of *w*. (*E*–*H*) Fixing *s* and instead varying sample size (*n*), we see that the magnitude of change as *w* increases grows with sample size (*n*), with the exception of expected allele frequency across all sites, which is independent of *n*. In plots shown, *σ* = 10, $\rho_{N}=20$, and $\mu =10^{-9}$.

As expected, the changes to the observed SFS with increasing sampling breadth have varying effects on downstream summary statistics (Fig. 4). Under our model, the expected allele frequency in the sample is equal to μ/s regardless of sampling strategy, including sample breadth and sample size (Fig. 4 *A* and *E*). This indicates that the discovery and dilution effects effectively cancel each other out, such that the average frequency (and in turn the expected heterozygosity) remains the same regardless of sampling (*SI Appendix*, Fig. S10).

However, other quantities of interest are indeed sensitive to the sampling breadth. First, we see that broader samples are expected to have a greater proportion of variant sites (Fig. 4 *C* and *G*). Second, the variant sites in broader samples are expected to have lower allele frequency (Fig. 4 *B* and *F*), as indicated by a higher fraction of singletons (Fig. 4 *D* and *H*) as compared to narrower samples. Together, these two results imply that broader samples will include more variants, but each variant will segregate at lower frequency on average. This result is consistent with intuition following the discovery and dilution effects described previously.

The exact behavior of these statistics with respect to *w* depends on the strength of selection, albeit weakly (Fig. 4*A*–*D*). With stronger selection, the observations converge to those expected under uniform sampling more rapidly as *w* is increased. When instead considering the values of expected summary statistics over the ratio $w/\ell_{c}$, we see that the rate and magnitude of change are consistent across selection coefficients (*SI Appendix*, Fig. S11). This is a result of the ratio $w/\ell_{c}$ being the key length scale in our model (Fig. 2*B*): For a fixed *w*, stronger selection reduces *ℓ*_*c*_ because allele carriers are more tightly clustered in space, and as a result, the sample is in effect more broad relative to the spatial dispersion of the carriers. Conversely, with the strength of selection held constant, increasing *w* results in the sample being more broad relative to the spatial dispersion of carriers.

Holding selection constant, we also see that the magnitude of effect as *w* increases becomes larger as *n* increases (Fig. 4*E*–*H*). These effects span several orders of magnitude.

### In- and Out-of-Model Simulations Validate the Results of Theoretical Analysis.

To validate our theoretical results, we performed two sets of population genetic simulations. First, we ran branching process simulations which correspond directly to our model. Inspecting the results, we see that the simulations and theoretical computations generally align for key outputs: the first two moments of the allele frequency (*SI Appendix*, Fig. S4), the joint parameters *λ*, *θ*_*E*_, and *γ*_*E*_ (Fig. 2), as well as the SFS itself (Fig. 3 and *SI Appendix*, Figs. S2 and S3). We observe some deviation from simulation results and theoretical expectations in two regimes (*SI Appendix*, Fig. S4): weaker selection regimes, in which we expect violations of the modeling assumptions regarding the rareness of the alleles and absence of homozygous carriers, and in regimes of very small sampling widths (*w* close to *σ*), which approach spatial scales where our measure-valued process may become a poor approximation to the reality of discrete individuals.

**Fig. 5.**
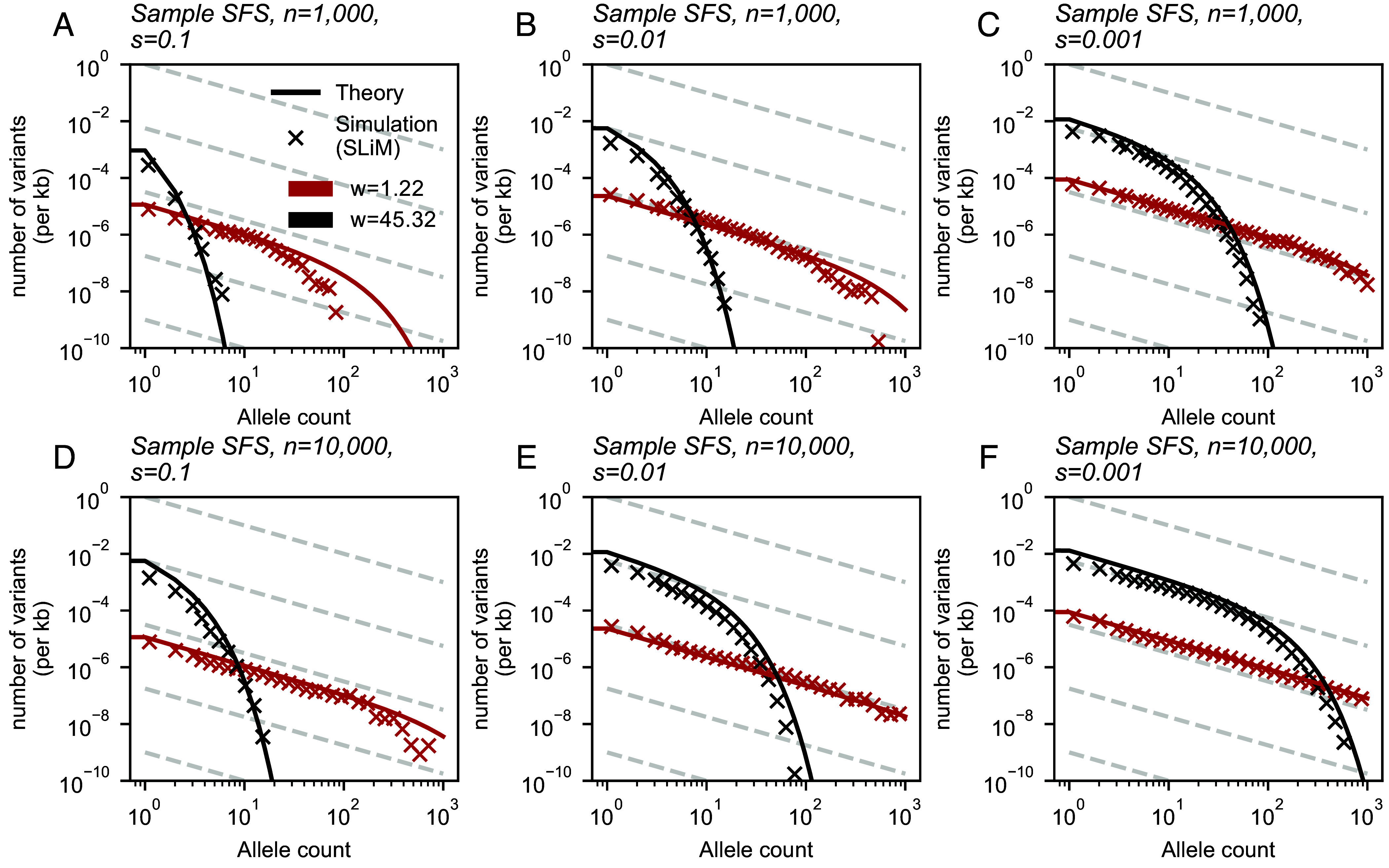
Simulations in SLiM compared to expected results under the model. SLiM simulations were performed using a modified version of the model of Battey et al. (42). After simulation, samples of size n=1,000 (*A*–*C*) and n=10,000 (*D*–*F*) were taken at varying sampling widths. Simulations shown were run using a habitat of width 75 units, population density of 5 individuals/unit squared, and deleterious mutation rate of $10^{-10}$ per basepair per generation. Frequency spectra shown are averaged over 100 sampling iterations. Parameters for theory results are directly matched to those of the simulations.

As a stronger test of the theory, we performed simulations in SLiM (48) using a modified version of the model of Battey et al. (42). These simulations are individual-based and do not make a number of the simplifying approximations used in our theoretical analysis. Fig. 5 shows the alignment between these simulations and our theoretical results across several parameter values. We generally see quite close alignment, with the exception of higher allele frequencies for the narrow sampling kernel (*w* = 1.22) in stronger selection regimes (*s* = 0.1) in which case our theory overestimates the number of variants relative to SLiM simulations.

These comparisons also reveal how computational efficiency varies greatly across the different approaches. SLiM simulation time ranged between 7.58 and 11.89 h (average: 8.64 h) per replicate for a habitat of length 75 units (50 replicates run per sample size and selection coefficient pair). On average, the branching process simulations completed in 18.59 min, 1.22 h, and 2.85 h per one million time steps for *s* = 0.1, 0.01, and 0.001, respectively for a habitat size of 10,000 units. In contrast, the time to generate theoretical frequency spectra shown in Fig. 3 ranged from 6.88 to 9.91 milliseconds per curve.

### Resampling Experiments in the UK Biobank Reveal Evidence of Discovery and Dilution Effects.

Having identified relationships between the spatial breadth of sampling effort and observed variant frequencies in our theoretical work, we now consider to what extent these patterns would arise in human genetic studies.

**Fig. 6.**
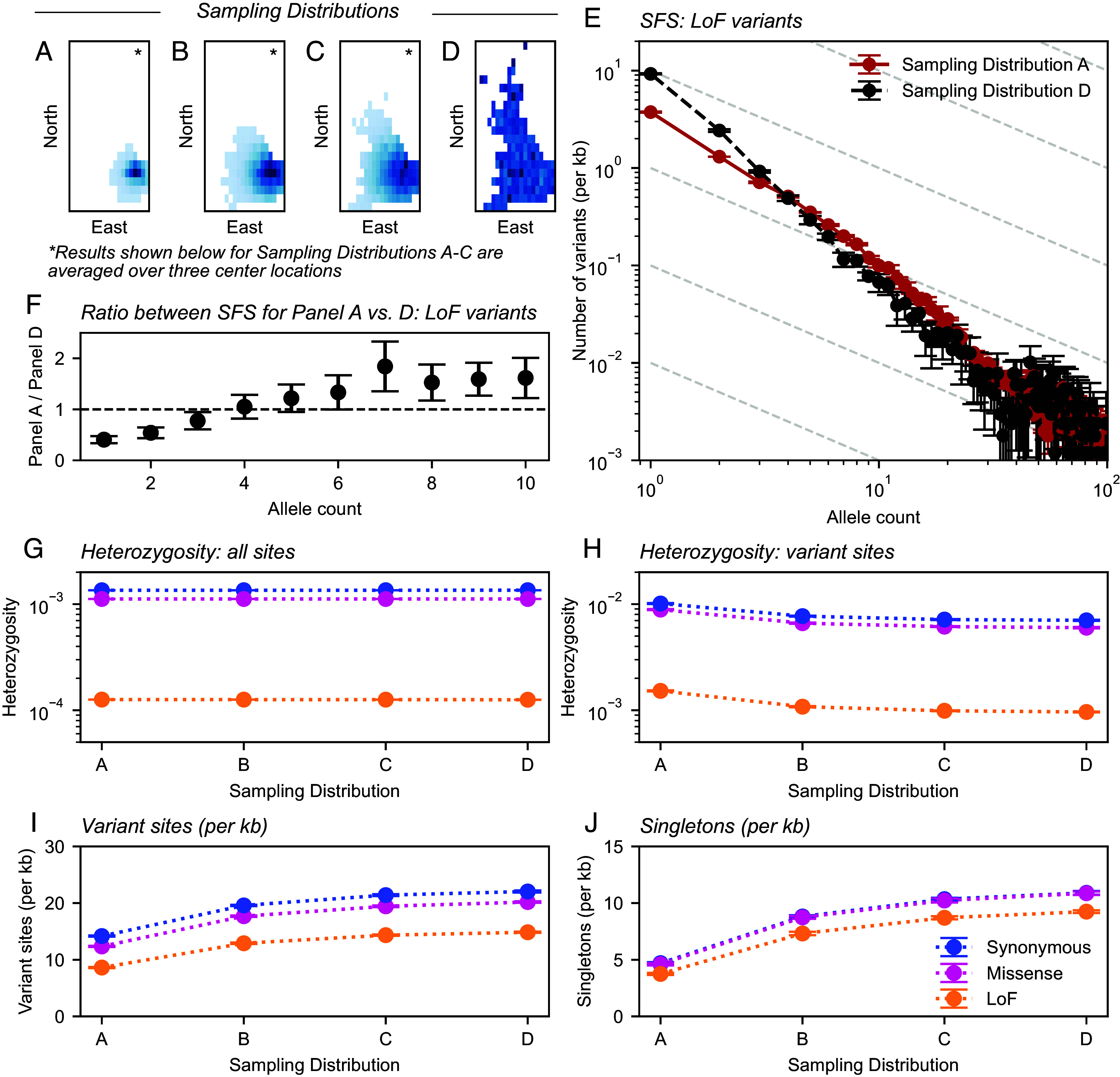
In silico resampling experiments in the UK Biobank using exome sequencing data from chromosome 1. Panels (*A*–*C*) depict outputs of the SIR procedure, where the distribution of birthplace coordinates follows a discretized Gaussian distribution. Sampling SDs (*w*), in order, are 50 km, 100 km, and 150 km. Gaussian sampling was repeated over three center locations (*SI Appendix*, Fig. S14) with averages across centers shown in other panels. Panel (*D*) depicts uniform sampling in this scheme. (*E*) Average folded sample SFS for putative LoF sites on chromosome 1 across sampling distributions, truncated at allele count of 100. (*F*) Ratio between SFS values between panels (*A* and *D*) for singletons through ten-tons. (*G*–*J*) Variation in summary statistics across sampling distributions and variant annotations. All results are averaged over ten sampling replicates with error bars representing two SEs.

To do so, we artificially mimic sampling designs that vary in geographic sampling breadth via in silico subsampling (n=10,000) of individuals from the large (n=469,835) exome sequencing dataset of the UKB, using sequencing data from Chromosome 1 (19). More specifically, we constructed samples spanning geographic scales of *w* = 50 km to uniform sampling based on birthplace data within the island of Britain for individuals with high genetic similarity to the centroid of the UK Biobank cohort (Fig. 6*A*–*D* and *SI Appendix*, Fig. S14). In constructing the samples, we adjusted for the underlying spatial heterogeneity in sampling density in the UKB using sampling importance resampling (*SI Appendix*, Fig. S13; 64).

Visualizing frequency spectra from these samples, we clearly observe differences in the SFS according to sampling breadth (Fig. 6 *E* and *F*). For instance, we observe an intersection between the SFS for uniform vs. narrow samples similar to that in our theoretical results (Fig. 6*E* and *SI Appendix*, Figs. S16 and S17). Additionally, when we compute the ratio of SFS values for the narrow sample (*w* = 50 km) vs. uniform sample, we identify a pattern similar to that seen in theory for rare alleles (counts less than 10; Fig. 6*F* and *SI Appendix*, Fig. S20).

Consistent with our theoretical predictions, as sampling scale becomes broader, one generally observes more variant sites, more singletons, and lower mean heterozygosity at variant sites, while mean heterozygosity across all sites is largely insensitive to changing sampling breadth (Fig. 6*G*–*J* and Table 1). The shifts in these summary statistics as sampling breadth increases are larger for the more putatively deleterious variant categories, though the differences are relatively small (Table 1). This is consistent with the theoretical results, which convey a dependence of the effects of sampling on the strength of selection that is relatively weak. The scale of the effects is such that for samples of size n=10,000, moving from *w* = 50 km to uniform sampling leads to on average 72.3% more discovered LoF variants per kb with a 36.75% reduction in heterozygosity at variant LoF sites (Table 1).

**Table 1.** Relative change in summary statistics between narrow (*w* = 50km) and uniform sampling distributions in birthplace (panels *A* and *D*, Fig. 6) based on resampling experiments across variant types

Variant type	*w* = 50 km	Uniform sampling	Relative change
Heterozygosity (all sites)			
Synonymous	$1.352\times 10^{-3}$	$1.354\times 10^{-3}$	0.15%
Missense	$1.120\times 10^{-3}$	$1.119\times 10^{-3}$	−0.09%
LoF	$1.260\times 10^{-4}$	$1.256\times 10^{-4}$	−0.32%
Heterozygosity (variant sites)			
Synonymous	$1.014\times 10^{-2}$	$7.038\times 10^{-3}$	−30.59%
Missense	$8.889\times 10^{-3}$	$5.999\times 10^{-3}$	−32.51%
LoF	$1.522\times 10^{-3}$	$9.626\times 10^{-4}$	−36.75%
Variant sites (per kb)			
Synonymous	14.18	22.05	55.5%
Missense	12.35	20.19	63.5%
LoF	8.62	14.85	72.3%
Singletons (per kb)			
Synonymous	4.70	10.91	132.1%
Missense	4.60	10.87	136.3%
LoF	3.75	9.25	146.7%

Results shown for sampling distribution *w* = 50 km are averaged over three center locations.

A notable deviation from our theoretical predictions is that we observe a convergence of the larger allele counts in the empirical SFS across different sampling strategies (Fig. 6*E* and *SI Appendix*, Figs. S16 and S17). We speculate this is due to how variants with larger allele counts in the narrow sample are old enough to have been broadly shared across the scale of Britain, as they possibly predate the genetic structure present in Britain and are thus plausibly less affected by sampling breadth.

As an additional in silico experiment, to emulate sampling on scales greater than the spatial scale of the island of Britain, we also construct subsamples across the UK Biobank at different scales of sampling breadth based on positions in PC1-PC2 space. Though the shift in the SFS is more subtle, we again find that as sampling breadth scale increases, the total number of variants increases, with a concomitant decrease in the heterozygosity of variant sites, such that the expected heterozygosity over all sites remains constant (*SI Appendix*, Fig. S15).

Finally, we carried out an exercise using maximum likelihood estimation to fit the *ρ*_*N*_, *σ*, and *s* parameters to see whether the simple model we assume can plausibly fit the data and what scales the fitted parameters would be (*SI Appendix*, Figs. S22 and S23). The fitted parameters $\hat{\sigma }\approx$ 21.37 to 51.92km, ${\hat{\rho }}_{N}\approx$ 0.31 to 1.53 per km^2^, $\hat{s_{M}}\approx$ 0.00702 for missense variants and $\hat{s_{L}}\approx$0.01 to 0.0119 for LoF variants are arguably within plausible ranges given historical estimates (see *SI Appendix*; 65, 66). However, ${\hat{\rho }}_{N}$ and $\hat{\sigma }$ are sensitive to the inclusion of singletons and doubletons, and the expected SFS using the best fit values still deviates from the observed SFS, suggesting the simple model should be extended for fitting the data more fully (e.g. to accommodate nonstationary population dynamics).

## Discussion

Here, we have addressed the question of how the geographic “breadth” of sampling effort in genetic studies impacts the discovery of rare, deleterious genetic variants using a theoretical approach. Our analysis shows how sampling affects the expected site frequency spectrum via both discovery and dilution effects: Geographically broad samples will discover a greater number of variants but each will be observed at lower allele frequencies than in narrow samples. In contrast, geographically narrow samples will include fewer variants, and these variants will appear concentrated in the sample, often at frequencies above what would be found in uniform samples.

In several ways, our results echo the impacts of sampling on neutral variation described in previous studies: Spatially broader samples tend to discover more variant sites overall; however, these alleles tend to be singletons and other low-frequency alleles (36–38, 42, 43, 67). Using our approach, we can directly account for and vary the strength of negative selection, and observe its effects on the frequency spectra (Figs. 3 and 5 and *SI Appendix*, Figs. S6 and S7) and summary statistics (Fig. 4) of selected alleles. The more deleterious a class of variants is, the smaller the spatial scale of their spread (*ℓ*_*c*_) will be, and in turn, the effects of increasing sample breadth saturate most quickly for more deleterious variants. That is, for more deleterious sites, the discovered alleles will be as diluted as they would be in a fully uniform sample at a comparatively smaller scale of sampling.

A notable outcome in the model is that expected allele frequency (and in turn expected heterozygosity) is constant with respect to sampling breadth. The result also holds empirically across several annotation categories in our analysis of the UK Biobank. For instance, with a sample size of 10,000, our experiments show broad resamples of the UK Biobank have on average 72.3% more variant sites (and 146.7% more singletons, for LoF variants), but 36.75% lower heterozygosity at variant sites than when samples are narrowly concentrated (Table 1 and Fig. 6), yet either sampling design will see an average heterozygosity of 0.013%. While perhaps not immediately obvious from observing how the SFS changes with sampling breadth, the result can be understood as an expected outcome of our model (assuming birth/death/mutation rates are constant and independent of geography) and other models of deleterious allele frequency evolution in the presence of variable demographic histories (68). Indeed, recent work by Stolyarova et al. (69) identifies similar patterns across sampling groups in the gnomAD dataset (5), and explains how the result is expected from considerations of how under mutation–selection balance, the average frequency of deleterious alleles is expected to be similar across populations.

Our results have important implications for two major areas of research that use observations of rare, deleterious variants: i) genetic association studies and ii) evolutionary genetic inferences of fitness effects. In the next paragraphs, we discuss our results within these two contexts.

In genetic association studies of disease phenotypes and complex traits, observed frequencies are intrinsically tied to statistical power. GWAS power is roughly linear in allele frequency for low frequency alleles, as x(1−x)≈x for small *x*, and the cumulative MAF that impacts power in burden tests is also directly dependent on the average allele frequency. While many studies have considered the impact of increasing sample size on power, our results suggest interesting trade-offs related to geographic sampling breadth. Discovering a greater number of variants in broader samples will expand the space of potentially identifiable associations. Yet, the dilution of allele frequency seen in broader samples will hinder power to detect an association to each particular variant using single-variant GWAS designs. These effects perfectly compensate for one another in terms of their effects on average allele frequency observed at deleterious sites, suggesting negligible impact overall on the rate of detecting phenotypic associations.

Such implications are tentative though—more in-depth analyses of the impacts on GWAS and burden test power are needed which consider factors not addressed by our model. Such factors include linkage disequilibrium patterns, corrections for population stratification, the increased rate of cryptic relatedness in narrow samples, increased variance due to nongenetic factors in broad samples, and the effects of recent human population growth. Furthermore, the question of how best to construct samples for human genetics research is intrinsically linked to discussions of representation in biomedical research (2, 3, 7). So, while the work here contributes insights into the impact of sampling on the SFS of discovered variants, we emphasize that sampling is only one element of the multifaceted challenge of study design.

A second area of research for which our results have key implications is the inference of fitness effects in evolutionary genetics. Many studies aim to infer the DFE from the frequencies of observed variants of different classes (22–25). Such studies often focus on the population-scaled selection coefficient (commonly, *Ns*) as the parameter of interest. Empirically, population genetic samples are typically taken from one or a few distinct locations, yet are modeled as a random sample from the total population. Our results imply that this practice will lead to biases in the inference of selection coefficients which will tend toward underestimating the strength of negative selection.

Specifically, we expect that sampling narrowly from a particular location will lead to artificially high (or “concentrated”) frequencies of deleterious variants. This can be readily seen in how the shape of the SFS becomes flatter for more narrow samples (Fig. 3 *A* and *B*). In terms of our theory, this corresponds to our result that for spatially concentrated sampling, the effective selection intensity, *γ*_*E*_, can be substantially less than *Ns*. In fact, values of *γ*_*E*_ are orders of magnitude lower than *Ns* within our test settings (Fig. 2). Thus, the frequencies used in the inference framework will be higher than expected under random mating, leading to biased estimates of *s*. This bias is likely to be most prominent for alleles under stronger selection, as the deviation of *γ*_*E*_ from *Ns* will be larger (Fig. 2). We also expect to see a downward bias in the inferred variance of the DFE for spatially concentrated samples: Estimates for variants with stronger fitness effects will be biased more strongly than those with weak effects, leading to an overall reduction in variability among inferred effects.

For both of these downstream applications, another relevant finding from our model is that the magnitude of effect of sampling width on allele frequencies and summary statistics is highly dependent on the sample size (Figs. 3 and 4*B* and *SI Appendix*, Fig. S5). Thus, as sample sizes in genetics continue to grow toward millions of individuals, we may expect the impact of sampling breadth to become more evident.

In relationship to other theoretical approaches to this problem, we note that previous analyses of spatial population genetic models have derived the spatial covariance between allele frequencies sampled at two locations (32–34 see *SI Appendix*). In retrospect, given our finding that second-order moments of the allele frequency distribution provide useful approximations of the SFS, an alternative route to obtain the results we obtain would be to use weighted averages of previously derived two-point spatial covariance functions to adjust for uneven sampling. The approach taken here has the advantage of being more quickly generalizable to higher-order moments (which can be used to refine the approximation), as well as to alternative spatial models of dispersal and sampling.

Indeed, the most important caveat of our work is that we analyze a highly abstract model of a spatial population and sampling effort. The deviations between the observed SFS and fitted SFS observed when we attempted to fit the model directly indicates deviations in the abundance of singletons and in how the SFS shifts more modestly with *w* than predicted (*SI Appendix*). We have also not considered various departures from equilibrium such as recent population growth, recent admixture from diverged lineages (e.g. archaic hominids), and recent origins from a shared ancestral population (e.g. shared African origins of humans). Also, our simple model of migration via local diffusion does not account for repeated layers of long-range dispersal events which are plausibly frequent in human and nonhuman populations (though such long-range dispersal can be approximated in the model using as an additional global homogenizing force similar to mutation, as done in Kimura and Weiss; 70). As a result, a geographically “narrow” sample in real data (e.g. sampling a city like London) are not truly “narrow” in the sense of our model. Also, our model also assumes that there are no boundaries on where carriers can disperse and as a result, no “boundary effects” are present. Thus, especially for settings beyond the UK Biobank, the relevance of these more complex factors should be kept in mind.

Overall, in real studies of populations of humans or other organisms, the patterns of movement and of sampling may greatly deviate from what we investigated here. Nonetheless, the general alignment of our empirical and theoretical results suggest the real-world importance of sampling breadth for interpreting the outcomes of existing studies and designing future ones.

## Supplementary Material

Appendix 01 (PDF)

## Data Availability

Some study data are available: All simulation data and associated scripts are available at: https://doi.org/10.5281/zenodo.15398319 (61). Empirical analyses used the published exome sequence data from the UK Biobank resource (19, https://www.ukbiobank.ac.uk/).
