# Supplementary material for: Study design and the sampling of deleterious rare variants in biobank-scale datasets
Source: Proc Natl Acad Sci U S A. 2025 Jun 3;122(23):e2425196122. doi: 10.1073/pnas.2425196122 (PMC12167998; doi:10.1073/pnas.2425196122)
Supplement: Supplementary file 1 — Appendix 01 (PDF) [file pnas.2425196122.sapp.pdf]

Supporting Information for

Study design and the sampling of deleterious rare variants in biobank-scale datasets

Margaret C. Steiner, Daniel P. Rice, Arjun Biddanda, Mariadaria K. Ianni-Ravn, Christian Porras, and John Novembre

Corresponding Author John Novembre.
E-mail: jnovembre@uchicago.edu

This PDF file includes:

Supporting text
Figs. S1 to S23
Table S1
SI References

Supporting Information Text

Extended theoretical methods

Here, we provide a detailed description of our theoretical methods. We model the movement, reproduction, and death of the carriers of a rare deleterious allele. These carriers are generated by mutations in a much larger population of wild-type individuals. By explicitly modeling only the rare carriers, we can approximate the evolution of their spatial distribution as a *superprocess*. We use the recent results of (1) to find the equilibrium moment generating functional of the spatial distribution of deleterious alleles at mutation-selection-migration-drift balance.Then, we apply a model of spatial sampling to compute the expected site frequency spectra for varying spatial sampling schemes.

In the following, we will assume that all parameters (i.e., population density, mutation rate, selection coefficient, and dispersal diffusion coefficient) are spatially and temporally homogeneous. It is straightforward to specify a more general model and to apply the same procedure we outline below to calculate its site frequency spectra. However, the numerical calculations become significantly more complicated. Therefore, we focus on getting intuition from the scaling results in the homogeneous case and leave generalizations to future work.

Population genetic model and relation to superprocesses. We consider a population of organisms living in a habitat H. Here we will focus on 2−dimensional continuous habitats, so that $H \subset \mathbb{R}^2$, but most of the theory applies to more general habitats. For simplicity, in our numerical calculations we will use a toroidal habitat of length L, i.e., $H = [-L/2, L/2]^2$ with periodic boundary conditions. Let ρ_N be the population density measure so that the number of individuals living in a region $A \subset H$ is given by $N(A) = \int_A \rho_N(\vec{x})dx$. The total population size is $N \equiv \int_H \rho_N(\vec{x})dx$. We assume that ρ_N is large and stably maintained by ecological forces so that we can neglect random fluctuations in population density due to migration, births, and deaths.

We are interested in tracking the number and spatial distribution of carriers of a rare deleterious variant with fitness cost $s > 0$. We will restrict ourselves to the weak-selection regime, $s \ll 1$. With this assumption, carriers of the deleterious allele have $1 - s$ offspring on average, compared to 1 for non-carriers. By focusing on rare alleles, we can neglect dominance effects because homozygous carriers should make up a negligible fraction of the carriers. We assume that wild-type alleles undergo mutation to the deleterious allele at rate μ per generation per individual. Thus, we model the influx of *de novo* mutations as a Poisson point process with intensity $\mu\rho_N(\vec{x})$. This approximation neglects the reduction in the mutation supply due to the fact that some fraction of the ρ_N might already carry the mutation. However, if $\mu \ll s$, the effect of selection dominates the resulting reduction in mutation supply and we can neglect it. We will similarly neglect back-mutation from the mutant to the wild-type.

For mathematical tractability, we will assume that carriers of the deleterious allele reproduce, die, and move about the habitat independent of one another and of the background of wild-type individuals. This assumption is justified as long as the deleterious allele remains rare. Given the rare-allele assumption, we model the movement of an individual carrier as a continuous-time Markov process on H with infinitesimal generator $\sigma^2\nabla^2$ (i.e., translation invariant, isotropic diffusion), independent of the positions of other carriers. The independence among copies in reproduction is equivalent to Haldane's branching process approximation to the Wright-Fisher process (2), and assuming independence of reproduction from geographic location has since been applied in spatial population genetic modeling (for instance, 3–5).

In a continuous habitat we need to choose a suitable definition of 'rare' that accounts for spatial variation in the mutant frequency. To wit, let σ be the standard deviation of the displacement of an individual in a single generation in each dimension. We assume that the number of carriers within a neighborhood of area $4\pi\sigma^2$ surrounding any particular individual is small compared to the total number of individuals in this region (i.e., $4\pi\rho_N\sigma^2$, Wright's neighborhood size, 6). As σ is the shortest length scale in the model, it is reasonable to assume that fluctuations in allele frequency at distances shorter than this scale will be short-lived and not contribute to the long-term evolution of the population.

Having defined the mutational process by which carriers are generated and the migration process by which they move around the habitat, it remains to define the mechanism by which they die and reproduce. Consistent with our assumption that carriers behave independently, we will model reproduction as a continuous-state branching process. Combining this with our Markov process for movement yields a superprocess model of the evolution of the spatial distribution of carriers (7, 8). For overviews of superprocesses and their properties, see (9, 10). If we add an influx of particles according to the mutational point process with intensity $\mu\rho_N$, we have a superprocess with immigration (11). (Confusingly, in the superprocess literature, our mutation process is called "immigration" and the migration process is sometimes called "mutation".) We now define this process and then give the main result (Eqs. 4–6) needed to calculate sample properties.

A superprocess $\{Z_t\}$ is a measure-valued random process. That is, $\{Z_t\}$ is a set of measures on the habitat H indexed by time, t. Measures are defined by how they integrate functions over their domain. Accordingly, we introduce the inner product $\langle Z, f\rangle \in \mathbb{R}$, defined as:

$$\langle Z, f\rangle = \int_H f(\vec{x})dZ(\vec{x}), \qquad [1]$$

where Z is a finite measure on H, and $f : H \to \mathbb{R}$ is a measurable function on H (9). The probability distribution of a superprocess at time t is characterized by its *moment generating functional* (MGF), Φ_t, defined as:

$$\Phi_t[f] = \mathbb{E}\left[\exp\left(\langle Z_t, f\rangle\right)\right]. \qquad [2]$$

Just as the derivatives of the moment generating function of a random variable gives its moments, the functional derivatives of Φ_t with respect to f give moments of the inner product $\langle Z_t, f\rangle$.

In our model, Z_t measures the number of carriers in a region of space. For a region $A \subseteq H$, we define an indicator function $I_A(\vec{x}) = 1$ for $\vec{x} \in A$ and zero otherwise, so that:

$$\langle Z_t, I_A\rangle = \text{count of carriers in } A, \text{ and } \langle Z_t, I_H\rangle = \text{total count of carriers.} \qquad [3]$$

By analogy, $\langle Z_t, f\rangle$, for an arbitrary non-negative f, gives the counts of carriers according to their positions by the weighting function f.

Friesen (1) has recently shown that a general set of subcritical superprocesses (i.e., ones where the measure decays exponentially) with immigration tend to a stationary distribution, subject to various technical conditions and they derive a moment generating functional for the density of the superprocess across the spatial domain. In particular, applying the Friesen result to our process with mutational supply intensity $\mu\rho_N$, diffusion coefficient σ^2, and selection coefficient s,

$$\lim_{t\to\infty} \Phi_t[f] = \Phi[f] \equiv \exp\left(\int_0^\infty \langle \mu\rho_N, u\rangle\, dt\right), \qquad [4]$$

where u_t is the solution to the semilinear PDE:

$$\frac{\partial}{\partial t} u(\vec{x}) = \sigma^2 \nabla^2 u - su + u^2, \qquad [5]$$

subject to initial condition:

$$u_0(\vec{x}) = f(\vec{x}). \qquad [6]$$

The function u does not have a direct biological interpretation, though its associated PDE incorporates several features of the evolutionary process (including selection and drift) . We note that more generally, the second order term (u^2) is multiplied by a factor of $a/2$ where a is the rate of increase in the variance in the number of descendants of one carrier (including itself). Here, as we scale time in terms of the baseline birth rate (1) and death rate (1) in the absence of selection, the value of a is 2 (approximately for small s; exact for $s = 0$) and so this factor simplifies to 1.

For our choice of time (scaling time in terms of the baseline birth/death rate without selection), the value of a is 2 (exactly for s=0, and approximately 2 for small s).

The stationary MGF, Φ, completely characterizes the counts and spatial distribution of carriers of the deleterious alleles of a population at steady-state. These patterns are due to the balance between the forces of mutation, selection, genetic drift, and migration. In the rest of this section, we will show how Eq. 4 can be used to calculate the expected site frequency spectrum for a spatially localized sample from the population.

Spatial sampling of the allele frequency distribution. We now connect the superprocess model of allele frequencies to the site frequency spectrum of a finite sample. We are interested in geographically biased samples, where the probability that an individual is sampled depends on its location according to a *sampling density*. Let the kernel function $g(\cdot)$ be the shape of the sampling density so that $\int_H g(x)dx = 1$. To capture the effect of broad versus narrow sampling, we will consider sampling kernels with a scale parameter w, which represents the typical distance between sampled individuals. In particular, for sampling on a torus, we will use a bivariate wrapped Gaussian sampling kernel with standard deviation w.

Note that the sampling density is a measure of the *a priori* sampling effort across space, rather than the realized locations of sampled individuals. In the following, we assume that we do not have access to the locations of our sampled individuals. However, this framework could be extended to consider the joint statistics of samples taken from multiple known locations.

We define the expected value of the k'th element of the site frequency spectrum (SFS) as the expected fraction of sites with k copies of the deleterious allele ($\xi_k^{(n)} = E[Pr(K = k)]$). Assuming that the samples are taken independently from the population with replacement, the number of copies of the deleterious allele is the sum of n Bernoulli trials with a random probability of success:

$$\xi_k^{(n)} = \mathbb{E}\left[\binom{n}{k} P^k (1-P)^{n-k}\right], \qquad [7]$$

where P is a random variable representing the probability any particular sampled allele is deleterious.

The distribution of P depends on (1) the locations of carrier individuals characterized by the superprocess Z, and (2) the probability that we sample a carrier given its location. For a sample taken uniformly from a region $A \subseteq H$, P is the fraction of carriers in A:

$$P(A) = \frac{\text{count of carriers in } A}{\text{total count of individuals in } A} = \frac{\langle Z, I_A\rangle}{N(A)}. \qquad [8]$$

If, instead, the sample is taken by choosing among a countable set of regions $\{A_i\}$ according to probabilities $\{g_i\}$ and then sampling uniformly within the chosen region,

$$P = \sum_i g_i \frac{\langle Z, I_{A_i} \rangle}{N(A_i)} = \left\langle Z, \sum_i \frac{g_i I_{A_i}}{N(A_i)} \right\rangle, \tag{9}$$

where the second equality uses the linearity of inner products. Taking the limit that sampling probabilities vary continuously according to a localized sampling density, we have

$$P \to \left\langle Z, \rho_N^{-1} g(\cdot) \right\rangle, \tag{10}$$

so that the sampling probability is an inner product of the random measure Z with the population-scaled sampling density.

Therefore, using Eqs. 2, 4, and 10, the moment generating function of P at steady-state is given by

$$\begin{aligned} \mathrm{MGF}_P(z) &\equiv \mathbb{E}\left[\exp\left(zP\right)\right] \\ &= \lim_{t\to\infty} \mathbb{E}\left[\exp\left(\left\langle Z_t, \rho_N^{-1} g(\cdot)\right\rangle\right)\right] \\ &= \Phi\left[\left(\frac{z}{\rho_N}\right) g(\cdot)\right] && [11] \\ &= \exp\left(\int_0^\infty \langle \mu\rho_N, u\rangle \, dt\right), && [12] \\ &= \exp\left(\mu\rho_N \int_0^\infty \int_H u(\vec{x}, t) d^2\vec{x} dt\right), && [13] \end{aligned}$$

where u_t solves Eq. 5 with initial condition

$$u_0(\vec{x}) = \left(\frac{z}{\rho_N}\right) g(\vec{x}). \tag{14}$$

In the following sections, we will use these equations to solve for key properties of the distribution of P in order to evaluate the SFS per Eq. 7.

Moments of the allele frequency distribution. Here, we aim to calculate the log of the MGF of P by way of Eq. 13. We consider a habitat $H = [-L/2, L/2]^2$ (for $L \in \mathbb{R}$) with periodic boundary conditions. For mathematical convenience, we will work in Fourier space. First, we take a Fourier transform of $u(\vec{x}, t)$ over both time and space:

$$\hat{u}(\vec{\nu}, \omega) \equiv \int_0^\infty \int_H u(\vec{x}, t) \exp\left(-2\pi i \left(\frac{\vec{\nu}\cdot\vec{x}}{L} + \omega t\right)\right) \frac{d^2\vec{x}}{L^2} dt \tag{15}$$

where $\omega \in \mathbb{R}$ (temporal frequency) and $\vec{\nu} \in \mathbb{Z}^2$ (spatial frequency). Applying the same transformation to the PDE in Eq. 5 gives:

$$\left(2\pi i\omega + \left(\frac{2\pi\sigma}{L}\vec{\nu}\right)^2 + s\right) \hat{u} = \hat{u} * \hat{u} + \frac{z}{\rho_N} \hat{g}(\vec{\nu}). \tag{16}$$

It then follows from Eqs. 13 and 15 that:

$$\log \mathrm{MGF}_P(z) = \mu\rho_N L^2 \hat{u}(\vec{0}, 0), \tag{17}$$

and as such we aim to solve for the value of $\hat{u}$ at the origin.

To proceed, we calculate a perturbative expansion of $\hat{u}$ in powers of z, up to the second order:

$$\hat{u} = z\hat{u}_1 + z^2\hat{u}_2 + \mathcal{O}(z^3). \tag{18}$$

Substituting into Eq. 16 gives:

$$\hat{u}_1 = \left(2\pi i\omega + \left(\frac{2\pi\sigma}{L}\vec{\nu}\right)^2 + s\right)^{-1} \frac{1}{\rho_N} \hat{g}(\vec{\nu}) \tag{19}$$

and

$$\hat{u}_2 = \left(2\pi i\omega + \left(\frac{2\pi\sigma}{L}\vec{\nu}\right)^2 + s\right)^{-1} \left(\hat{u}_1 * \hat{u}_1\right). \tag{20}$$

Evaluating at the origin, we have:

$$\hat{u}_1(\vec{0}, 0) = \frac{\hat{g}(\vec{0})}{s\rho_N} = \frac{1}{s\rho_N L^2} \tag{21}$$

and

$$\hat{u}_2(\vec{0},0) = s^{-1}\left(\hat{u}_1 * \hat{u}_1\right)(\vec{0},0) \quad [22]$$

$$= s^{-1}\sum_{\vec{\nu}}\left(\int_{-\infty}^{\infty}\frac{1}{(2\pi\omega)^2+\left(\left(\frac{2\pi\sigma}{L}\vec{\nu}\right)^2+s\right)^2}d\omega\right)\frac{\hat{g}(\vec{\nu})\hat{g}(-\vec{\nu})}{{\rho_N}^2} \quad [23]$$

$$= \frac{1}{2s^2{\rho_N}^2}\sum_{\vec{\nu}\in\mathbb{Z}^2}\frac{\hat{g}(\vec{\nu})\hat{g}(-\vec{\nu})}{\left(\frac{2\pi\ell_c}{L}\vec{\nu}\right)^2+1}, \quad [24]$$

where the last line introduces the critical distance $\ell_c = \sqrt{\frac{\sigma^2}{s}}$. We refer to this value as the *characteristic length scale* (see section 3.1 in the main text).

From here, we can calculate the mean and variance of P. We find that the mean allele frequency is independent of the sampling scheme:

$$\mathbb{E}[P] = \frac{d}{dz}MGF_P\Big|_{z=0} \quad [25]$$

$$= \mu\rho_N L^2\hat{u}_1(\vec{0},0) \quad [26]$$

$$= \frac{\mu}{s}. \quad [27]$$

On the other hand, the variance is given by:

$$\mathbb{V}[P] = 2\mu\rho_N L^2\hat{u}_2(\vec{0},0) \quad [28]$$

$$= \frac{\mu}{s^2\rho_N L^2}\sum_{\vec{\nu}\in\mathbb{Z}^2}\frac{L^4\hat{g}(\vec{\nu})\hat{g}(-\vec{\nu})}{\left(\frac{2\pi\ell_c}{L}\vec{\nu}\right)^2+1}, \quad [29]$$

and so is dependent on the sampling kernel $g(\cdot)$. If sampling is uniform over the habitat, $\hat{g}(\vec{\nu}) = L^{-2}\delta_{\vec{\nu}}$. Thus,

$$\mathbb{V}[P] = \frac{\mu}{s^2\rho_N L^2} = \frac{\mu}{s^2 N}. \quad [30]$$

On the other hand, if sampling follows a wrapped normal distribution with scale w,

$$\mathbb{V}[P] = \frac{\mu}{s^2\rho_N L^2}\sum_{\vec{\nu}\in\mathbb{Z}^2}\frac{\exp\left(-\left(\frac{2\pi w}{L}\vec{\nu}\right)^2\right)}{\left(\frac{2\pi\ell_c}{L}\vec{\nu}\right)^2+1}. \quad [31]$$

For $w \gtrsim L$, the numerator of the sum falls off rapidly with $|\vec{\nu}|$ and we converge to the uniform sampling result. For $\ell_c \gg L$, the denominator grows large for $|\vec{\nu}| > 0$ and we again converge to the uniform sampling result. For $w, \ell_c \ll L$, we can approximate the sum with an integral:

$$\mathbb{V}[P] \approx \frac{\mu}{s^2\rho_N}\int_{\mathbb{R}^2}\frac{\exp\left(-\left(\frac{2\pi w}{L}\vec{\nu}\right)^2\right)}{\left(\frac{2\pi\ell_c}{L}\vec{\nu}\right)^2+1}\frac{d^2\vec{\nu}}{L^2} \quad [32]$$

$$= \frac{\mu}{s^2\rho_N 4\pi\ell_c^2}\exp\left((w/\ell_c)^2\right)E_1\left((w/\ell_c)^2\right). \quad [33]$$

Here, E_1 refers to the exponential integral function, defined as $E_1(x) \equiv \int_1^\infty (e^{-tx}dt)/t$.

For $w \gg l_c$, this is approximately $\mu/(s^2\rho_N 4\pi w^2)$, which implies that we converge to the uniform sampling result when $w \approx L/(2\sqrt{\pi}) \approx 0.3L$. For $w \to 0$, the expression diverges as we integrate over very large frequencies, which correspond to very small length scales where our model breaks down. We can remedy this by imposing a cutoff on $|\vec{\nu}|$. The smallest length scale our model can sensibly talk about is σ, the per-generation dispersal distance. Thus, a reasonable cutoff is $|\vec{\nu}| = L/\sigma$.

We validate these expressions by comparing results for $\mathbb{E}[P]$ and $\mathbb{E}[P^2]$ in simulations to their respective values under the model, and find a close correspondence (Fig. S4). Additionally, as the sampling width approaches the habitat width in the simulations, values of $\mathbb{E}[P^2]$ approach expected values under uniform sampling, as expected.

We note that Eqns. 24 and 29 are related to the two-point spatial covariance functions calculated in previous analyses of models of neutral (e.g. 12–14) or deleterious alleles (15, 16) that do not account for uneven sampling. We refer the reader to Eq. 4.40 in (14), Eq. 16 in (15), and Eqns. 22 and 32 in (16). Since our investigation has shown that the first two moments of the allele frequency distribution are sufficient to approximate the full SFS, it is more clear now that an alternative derivation of our Eqns. 24 and 29 can be obtained by averaging the existing two-point covariance functions from previous theory over spatial distance intervals and weighting those intervals appropriately by the spatial sampling density function. However, the methods for the derivation shown here are immediately generalizable to alternative dispersal functions and higher order moments (which can be used to calculate corrections to the Gamma distribution in cases where two moments do not provide a good approximation).

Effective parameters and the sample SFS. Having calculated the first two moments of P, we will now use them to approximate the full distribution of P and use this to calculate the SFS of a sample of finite size. We will assume that P approximately follows a Gamma distribution whose parameters we can calculate from the first two moments. If sampling is uniform over the habitat, this holds exactly (see section), and we show via simulation that this assumption is reasonable for non-uniform sampling as well (Figs. S2 and S3). Thus, we assume P follows:

$$P \sim \text{Gamma}(\theta_E, \gamma_E), \tag{34}$$

and we will refer to the shape and rate parameters, θ_E and γ_E, as the *effective mutation supply* and *effective selection intensity*, respectively. The motivation behind these names will be clarified by their derivation.

We derive the form of these parameters for both the uniform sampling and wrapped normal sampling cases using the method of moments applied to previous results (Eqs. 27-33). When sampling is spatially uniform, we have:

$$\theta_E = \frac{\mu^2/s^2}{\mu/(s^2 N)} = \mu N, \tag{35}$$

and

$$\gamma_E = \frac{\mu/s}{\mu/(s^2 N)} = sN. \tag{36}$$

When sampling follows a wrapped normal distribution with scale w, we have:

$$\begin{aligned}\theta_E &= \frac{\mu^2/s^2}{(\mu/(s^2\rho_N 4\pi\ell_c^2))\exp\left((w/\ell_c)^2\right)E_1\left((w/\ell_c)^2\right)} \\ &= \mu\frac{4\pi\rho_N\ell_c^2}{\exp((w/\ell_c)^2)E_1((w/\ell_c)^2)} &[37]\\ &= \mu\rho_N\ell_c^2\lambda, &[38]\end{aligned}$$

where:

$$\lambda \equiv \frac{4\pi}{\exp((w/\ell_c)^2)E_1((w/\ell_c)^2)}. \tag{39}$$

Similarly, we have:

$$\begin{aligned}\gamma_E &= \frac{\mu/s}{(\mu/(s^2\rho_N 4\pi\ell_c^2))\exp\left((w/\ell_c)^2\right)E_1\left((w/\ell_c)^2\right)} \\ &= s\frac{4\pi\rho_N\ell_c^2}{\exp((w/\ell_c)^2)E_1((w/\ell_c)^2)} &[40]\\ &= s\rho_N\ell_c^2\lambda. &[41]\end{aligned}$$

The compound scale factor λ (which we refer to as the *sampling effect scalar*) captures all spatial sampling aspects of the problem.

To summarize, we define the distribution of P for the uniform sampling case as:

$$P \sim \text{Gamma}(\mu N, sN), \tag{42}$$

and for the wrapped Normal case as:

$$P \sim \text{Gamma}(\mu\rho_N\ell_c^2\lambda, s\rho_N\ell_c^2\lambda). \tag{43}$$

For a sample of n haploid genomes, let $K \in \{0, \ldots, n\}$ be a random variable representing the number of sampled copies of the deleterious allele in the focal site. Recall from Eq. 7 that the number of copies of the deleterious allele is the sum of n Bernoulli trials with probability of success P, or equivalently, $K \sim \text{Binom}(n, P)$. For large n and small P, this is approximately $K \sim \text{Pois}(nP)$. Then, from properties of Gamma-Poisson mixtures, allele counts in a finite sample of size n follow:

$$K \sim \text{NegBin}\left(\mu N, \frac{sN}{sN+n}\right), \tag{44}$$

for the uniform sampling case, and:

$$K \sim \text{NegBin}\left(\mu\rho_N\ell_c^2\lambda, \frac{s\rho_N\ell_c^2\lambda}{s\rho_N\ell_c^2\lambda + n}\right), \tag{45}$$

for the wrapped Normal sampling case. We can use these distribution to calculate elements of the SFS as:

$$\xi_k^{(n)} \equiv \Pr\{K = k\}. \tag{46}$$

The remainder of the results follow from these expressions.

Derivation of expected values for summary statistics of the SFS. Having derived the form of the SFS, we can now obtain expressions for various population genetic summary statistics. We will show explicit derivations only in the case of wrapped Normal sampling, for brevity, though similar derivations can be obtained easily for the uniform sampling case. First, we consider the expected proportion of variant sites in a sample, or equivalently the probability that a particular allele segregates in a sample of size n. This follows from Eq. 45:

$$\begin{aligned}\Pr\{K>0\} &= 1-\Pr\{K=0\}\\ &= 1-\left(\frac{\gamma_E}{\gamma_E+n}\right)^{\theta_E}\end{aligned}\qquad [47]$$

Mean allele frequency is invariant to the scale of sampling:

$$\begin{aligned}\mathbb{E}\left[\frac{K}{n}\right] &= \frac{1}{n}\cdot\theta_E\cdot\frac{n}{\gamma_E+n}\cdot\frac{\gamma_E+n}{\gamma_E}\\ &= \frac{\theta_E}{\gamma_E}\\ &= \frac{\mu}{s},\end{aligned}\qquad [48]$$

though the mean frequency of *non-monomorphic* alleles does vary according to the sampling design. We obtain an expression for the conditional mean as follows:

$$\begin{aligned}\mathbb{E}\left[\frac{K}{n}\middle|K>0\right] &= \frac{1}{n}\sum_k k\frac{\Pr(\{K=k\}\cap\{K>0\})}{\Pr\{K>0\}}\\ &= \frac{\mathbb{E}[K/n]}{\Pr\{K>0\}}\\ &= \frac{\mu/s}{1-\left(\frac{\gamma_E}{\gamma_E+n}\right)^{\theta_E}}.\end{aligned}\qquad [49]$$

Heterozygosity is defined as the probability that two alleles are different from one another, and so it follows from the distribution of P rather than the SFS. Accordingly, we calculate expected heterozygosity as follows, using Eq. 34:

$$\mathbb{E}[2P(1-P)] = 2\mathbb{E}[P]-2\left[\mathbb{V}[P]+\mathbb{E}[P]^2\right]\qquad [50]$$

$$= \frac{2\theta_E}{\gamma_E}\left[1-\left(\frac{1-\theta_E}{\gamma_E}\right)\right].\qquad [51]$$

Exact solution for the uniform sampling case. Here, we provide an exact derivation of the distribution of P when sampling is uniform, providing motivation for our approximation that P is approximately Gamma-distributed more generally. Eq. 5 is a nonlinear parabolic PDE and can not be solved in closed form for general initial conditions. However, if we sample uniformly over the habitat, $u_0(x)$ becomes constant and $\nabla^2 u = 0$, yielding a Bernoulli ODE for u, which we can solve exactly. Note that in our model, uniform sampling is equivalent to sampling from a panmictic population. This is because we are focused on rare alleles, which are assumed not to interact. For common alleles, local fixation changes the dynamics qualitatively and breaks this equivalence.

For uniform sampling, $g(x) = 1/L^2$, and the spatial derivative term vanishes so that Eqns. 5, 13, and 14 become:

$$\frac{d}{dt}u = -su+u^2\qquad [52]$$

$$\mathrm{MGF}_P(z) = \exp\left(\mu N\int_0^\infty u(t)dt\right)\qquad [53]$$

$$u(0) = \frac{z}{\rho_N L^2} = \frac{z}{N}\qquad [54]$$

At this point, we could solve Eq. 52 directly. Instead, we will motivate our approach to the non-uniform sampling case by finding a power series solution for u: $u(t) = \sum_{k=0}^{\infty} z^k u_k(t)$. Substituting into Eq. 52 and organizing the terms by powers of z, we can generate an infinite sequence of ODEs for the terms $\{u_k\}$. Starting with $k=0$, we have

$$\frac{d}{dt}u_0 + su_0 = u_0^2,\qquad [55]$$

with initial condition $u_0(0) = 0$. This has the trivial solution $u_0 = 0$.

For $k > 0$, we have

$$\frac{d}{dt}u_k + su_k = \sum_{\ell=1}^{k-1} u_\ell u_{k-\ell} \qquad [56]$$

$$u_k(0) = \begin{cases} \frac{1}{N}, & k = 1 \\ 0, & \text{otherwise.} \end{cases} \qquad [57]$$

Each equation in the hierarchy is a first-order linear ODE with a forcing term that depends only on the solutions to lower-order terms.

Then it can be shown by induction that for all $k \geq 1$, the following holds:

$$u_k = N^{-k} \left(\frac{1 - e^{-st}}{s} \right)^{k-1} e^{-st}. \qquad [58]$$

Then, by property of a geometric series:

$$u(t) = \sum_{k=0}^{\infty} z^k u_k = \frac{\frac{z}{N} e^{-st}}{1 - \frac{z}{sN}(1 - e^{-st})}. \qquad [59]$$

Substituting into equation Eq. 53 gives:

$$\log \mathrm{MGF}_P(z) = \mu N \int_0^\infty \frac{\frac{z}{N} e^{-st}}{1 - \frac{z}{sN}(1 - e^{-st})} dt \qquad [60]$$

$$= -\mu N \log \left(1 - \frac{z}{sN} \right), \qquad [61]$$

and so:

$$\mathrm{MGF}_P(z) = \left(1 - \frac{z}{sN} \right)^{-\mu N}. \qquad [62]$$

Thus, P follows a Gamma distribution with rate sN and shape μN, which is consistent with predictions from classical population genetic models (17, 18). The equality of the uniform sampling result just derived with the classical results from a panmictic population is implicit in the assuming events in the branching process occur independently of geographic location, however the derivation shown here provides an introduction to the PDE-based approach we take when modeling uneven sampling. It also foreshadows our results that the form of the SFS is approximated by by two compound parameters representing selection and mutation, respectively.

Extended simulation methods

Here, we provide details on our simulation methods. All simulation code and associated scripts are available at: https://github.com/NovembreLab/spatial_rare_alleles.

Spatial branching process simulations. Our first set of simulations is based on a branching process framework and aligns closely with our theoretical model. The habitat is a square of length L with periodic boundary conditions. Consistent with our theoretical model, carriers appear *de novo* with rate $\mu \cdot \rho_N$, give birth with rate $1 - s$, and die at rate 1. Between events, dispersal of individuals occurs according to a Gaussian distribution with variance $\sigma^2 t$ where t is the time between events. We sample alleles at random times at rate r, according to a wrapped Gaussian sampling kernel with scale parameter w. We implement these simulations via the Gillespie algorithm (19). For computational efficiency, we implement a form of pseudo-replication in that for each simulation, we sample 100 evenly-spaced sampling centers within the habitat. Each simulation runs for 10 million generations.

Initially, we use these simulations to confirm that a negative binomial PMF provides a good fit to the simulated SFS via the method of moments (Fig. S2-S3). The ouput of each simulation is a vector of sampled values of P, which we use to calculate the first two moments of the allele frequency distribution: $\mathbb{E}[P]$ and $\mathbb{E}[P^2]$ (Fig. S4). Having computed these moments, we can then calculate the key parameters of the expected SFS under our model (equivalently to Eq. 38 and Eq. 41):

$$\theta_E = \frac{\mathbb{E}[P]^2}{V[P]}, \qquad [63]$$

and

$$\gamma_E = \frac{\mathbb{E}[P]}{V[P]}. \qquad [64]$$

We can then use ratios between these terms to compute the λ parameter.

SLiM simulations. Our second set of simulations implements a previously developed spatial model (20) in SLiM (21). All conditions are the same as in Battey et al. (20) except that all variants are deleterious with some selection coefficient. For each selection coefficient and sample size of interest, we run 50 replicates of the simulation in a square habitat 75 units wide with a population density of 5 for a genome of length 100 Mbp and mutation rate 1×10^{-10} per base pair per generation. The dispersal distance (equivalent to our σ parameter) is set to 0.2 units. For each simulation run, we sample individuals according Gaussian (with varying width) or uniform distributions and obtain the sample SFS. We then average over 100 sampling iterations for each width.

The model from Battey et al. (20) includes several factors that are not modeled in our theory or branching process simulations. For instance, their model includes non-toroidal boundary conditions, with the probability of individual survival declining near range edges to avoid upward biases in fitness. A particularly notable difference in the two models is the definition of the parent-offspring distribution. In our model, individuals disperse away from their location of origin (which is a single point) at root mean squared distance σ per generation. Under the Battey et al. model, individuals arise as the offspring of two parents, and dispersal occurs from each parent 50% of the time. This results in a constant scalar difference in all spatial parameters between the Battey et al. model and ours (under our simulation parameters, this scalar works out to 4.08). However, we find that since both w and σ parameters in our model are scaled by this factor, it cancels out of w/ℓ_c and thus λ as well as $\rho\ell_c^2$. As a result, estimates of θ_E and γ_E (and thus, all downstream results) are not affected by this difference. We note that time-scales for mutation are the same between models (per-generation).

Extended empirical methods

Here, we provide additional detail on our empirical methods for analyses described in the main text. Scripts and executables are available at: https://github.com/NovembreLab/spatial_rare_alleles.

Sampling importance resampling algorithm and implementation. A key step in our empirical analysis is to construct samples within the UK Biobank having Gaussian or uniform distribution. Here, we provide detail on the sampling procedure used.

For samples of individuals in geographic (birthplace) space, we first filter the data to individuals passing QC (per UK Biobank metrics 22), born in the UK, with coordinates available, and having Euclidean distance within 0.0001 of the median centroid in PC1-PC2 space. We then calculate binned frequencies of birthplaces in discretized geographic space (20x20 grid).

For uniform samples, we assign weights that are inversely proportional to the frequency of individuals in the bin in which an individual lies. For Gaussian samples, we compute distance per-individual from one of three pre-selected center points (located centrally within a bin) and assign weights according to a Gaussian density with standard deviation w, divided by the binned frequency as used in the uniform weight calculation. Centers were chosen to avoid known urban or otherwise high-density areas (to avoid model mis-specification) and such that center locations had sufficient individuals in the nearby region as to avoid extreme re-sampling of individuals. For samples in PCA space, this process is identical except that we use all individuals passing QC, construct the 20x20 grid over PC1-PC2 space, and only use one center point (which corresponds to the bin including the median value). All weights are normalized to sum to one.

Then, using custom scripts, for each set of weights we sample sets $10,000$ individuals *with replacement*. As a result of the weighting scheme used and this sampling step, the birthplace locations/PC1-PC2 coordinates for each sample match either a uniform distribution or a Gaussian distribution with weight w, as intended (see, for instance, Fig. S14). We then compute and output the SFS for each sample. We limit our analysis to single nucleotide variants (SNVs) on chromosome 1.

Scaling of SFS values. In order to report the SFS on a per-kilobase scale, we adjust output values to account for both the proportion of SNVs kept in the procedure to downsample to a common sets of 32,320 variants (13.1%, 6.4%, and 100% for synonymous, missense, and LoF variants, respectively) and the total length of exonic sequence which has the potential to mutate to produce a variant of each type. For synonymous and missense variants, this is assumed to be 1/3 and 2/3 of the total length of sequenced exons on chromosome 1 (as determined by sequencing target regions provided by UKB). For LoF variants, this is computed as the total number of sites of potential LoF mutations as identified by Zeng et al. (23) which overlap with sequencing targets on chromosome 1. This results in the following values (kb) of sequence length for synonymous, missense, and LoF variants, respectively: 1,308 kb, 2,616 kb, and 167.6 kb. Together, this suggests output should be divided by the quantity (length of exonic sequence) * (proportion of SNVs kept). These corrections are applied to the SFS as well as derivative quantities (number of singletons and segregating sites).

Calculation of summary statistics. Having computed the SFS, we then compute various summary statistics. The number of variant sites, number of singletons are obtained by simple counting from the SFS values obtained after SIR sampling.

For computing mean allele frequencies per basepair of sequence data, we must account for sites that are monomorphic in the UKB sample and that we sub-sampled all functional categories to $L_S = 32,320$ (the number of LoF variants on chromosome 1) to make comparisons across frequency categories simpler (and to simplify the computational burden of the SIR sampling). In brief, for each functional category the mean frequency $\bar{p}$ we aim to compute can be expressed as:

$$\bar{p} = \frac{1}{L_T}\left(\sum_{i=1}^{L_V} p_i + \sum_{i=1}^{L_M} p_i\right) \quad [65]$$

with L_T as the total number of sites of the given functional category , L_V as the number of those sites observed as variable in UKB, and L_M as the number of those sites monomorphic in UKB. From the data, we calculate the mean across L_S which is a fraction c of the L_V sites variable in UKB, $\bar{p}_S = \frac{1}{L_S}\sum_i^{L_S} p_i$. Given the p_i are necessarily 0 for all L_M monomorphic sites and $L_S\bar{p}_S/c$ is a moment-based estimator of $\sum_i^{L_V} p_i$, we can estimate $\bar{p}$ as:

$$\bar{p} = \frac{L_S}{cL_T} \cdot \bar{p}_S. \qquad [66]$$

Across all three variant classes, we found the ratio $\frac{L_S}{cL_T}$ was 0.19 in our data.

Computations of average allele frequencies per annotation category were corrected for reference bias in the annotation categories following the procedure described by Simons et al. (24). In brief, SNVs for which the reference allele is derived were assumed to be synonymous, missense, and LoF at identical rates to variants for which the reference allele is ancestral (within a frequency bin). The average frequency for each variant type thus includes all ancestral-reference alleles of that type as well as weighted contributions from derived-reference alleles. Ancestral vs. derived alleles were determined using the ancestral reference provided by Ensembl release 112.

For computing expected heterozygosity, to incorporate the annotation-reference bias correction, we approximate the expected heterozygosity of the data as two times the bias-correeected average minor allele frequency.

All results shown are averaged over ten SIR replicates each, and values are reported per-kb as appropriate. Additionally, metrics for samples within geographic (birthplace) space are averaged over each of the three sampling centers.

Supplemental analyses

Here, we summarize methods and results for additional empirical analyses using the UK Biobank data. Scripts and executables are available at: https://github.com/NovembreLab/spatial_rare_alleles.

Maximum likelihood fitting of parameter values. While the modeling we undertook here is primarily intended to improve an understanding of the conceptual role of various parameters that impact sampling breadth, rather than for the purposes of precisely modeling human genetic variation, we undertook an exercise fitting the model to the UK Biobank data. To do so, we fixed $\mu = 1.25 \times 10^{-8}$ and $w \in$[50km, 100km, 150km] as appropriate, and estimated ρ_N and σ and s using maximum likelihood estimation (MLE) via a grid search. For the grid search, we evaluated $\sigma \in [1, 500]$ km (50 linearly-spaced values), $\rho \in [1 \times 10^{-4}, 500]$ per km^2 (50 linearly-spaced values), and $s \in [1 \times 10^{-4}, 1 \times 10^{-1}]$ (40 log-spaced values).

To compute a likelihood for the parameters, we use a Poisson Random Field approach and assume the counts of variants at each discrete allele frequency are independent Poisson random variables with a mean given by our theory (Eq. 46 multiplied by the appropriate number of basepairs). We compute the likelihood using as input the counts ranging from singletons to 10-tons as we do not expect the theory to fit for larger allele counts both due to departures from the rare allele assumptions, and the concomitant inclusion of variants that are older and hence increasingly likely to have experienced non-diffusive dispersal dynamics not captured in the model. We also computed the liklelihood excluding singletons as well as excluding singletons and doubletons, as these values in the data may be more error-prone (from sequencing artifacts) or influenced by recent rapid human population growth not captured by the model. Given their higher observed numbers, we focused on the missense variants as input for obtaining estimates of $\hat{\sigma}$ and $\hat{\rho_N}$ and $\hat{s_M}$ (the selection coefficient for missense variants). As a second step, we fixed σ and ρ_N to $\hat{\sigma}$ and $\hat{\rho_N}$, and obtained an MLE $\hat{s_L}$ of the selection coefficient for the LoF variants.

We find plausible maximum-likelihood combinations of σ, ρ_N, s_M that put the expected counts on a similar scale to that observed in the empirical frequency spectra ($\hat{\sigma} \approx$ 21.37-51.92km, $\hat{\rho}_N \approx$ 0.31-1.53 per km^2, $\hat{s}_M \approx 0.00702$ for missense variants and $\hat{s}_L \approx$0.01-0.0119 for LoF variants; Figs. S22-S23). We note that the values of $\hat{s}_L$ for LoF variants are within a plausible range for humans, given results of Zeng et al. (23). Further, the shift in the SFS towards increasing lower frequency variants for larger w is approximately captured by the theory.

Qualitatively, we see two deviations from our theory. First, our theory predicts a stronger shift in the SFS with response to changes in w than is observed in data (note how in the figure the theory lines shift more than the underlying data do). We expect this may be due to more effective mixing across geographic space than implied by the diffusive model we assume in our theory. Second, the results are sensitive to the inclusion of singletons and doubletons, such that maximum likelihood parameters fit on tripletons to 10-tons correspond to frequency spectra that under-estimate singleton and doubleton counts. We expect that this sensitivity may be due to our theory not incorporating recent patterns of human population growth. In all cases, the fit is worse outside of the range of fitted counts (especially higher count values), likely due to violations from the equilibrium assumptions of our model.

PCA-based sub-sampling. As means to approximate the effects of sampling on broader geographic scales than the island of Britain, we explored a PCA-based approach in which we treat PC1-PC2 coordinates as proxies for geographic location. This approach is motivated in part by how participants in the biobank as a whole span a broad range of ancestral backgrounds (22) and previous results on how coordinates in PC1-PC2 space approximate geographic distance (25, 26). For this approach, we center the distribution of sample (PC1, PC2) coordinates at the centroid of PC1-PC2 space and construct samples of size $n = 10,000$ from various sampling breadths w of 0.0015, 0.0025, and 0.005 using a Gaussian sampling density with standard deviation w, as well as a uniform sample (panels E-H in Fig. S15). We repeat the sampling procedure ten times for each sampling width.

Trends in summary statistic values broadly follow patterns we expect from our theoretical results and observed in the main empirical results (Fig. 4, Table S1;Fig. S15). Moving from $w = 0.0015$ to uniform sampling leads to 15.02% more LoF variants per kb with a 4.27% reduction in heterozygosity at variant LoF sites (Table S1). As expected, the SFS for the narrowest samples shows fewer variants total, fewer variants at low frequencies, and an enrichment of variants found at higher counts (particularly in counts >100). Analogous results for synonymous and missense variants are provided in Table S1.

Relative to our main results, the results of this experiment on the impact of sampling are much more subtle. This is possibly due to some issues with using PCA-space as substitute for geographic space. It also may be that at these implicitly broader scales w is large enough that the effects of w essentially saturate (e.g. as in Fig. 4).

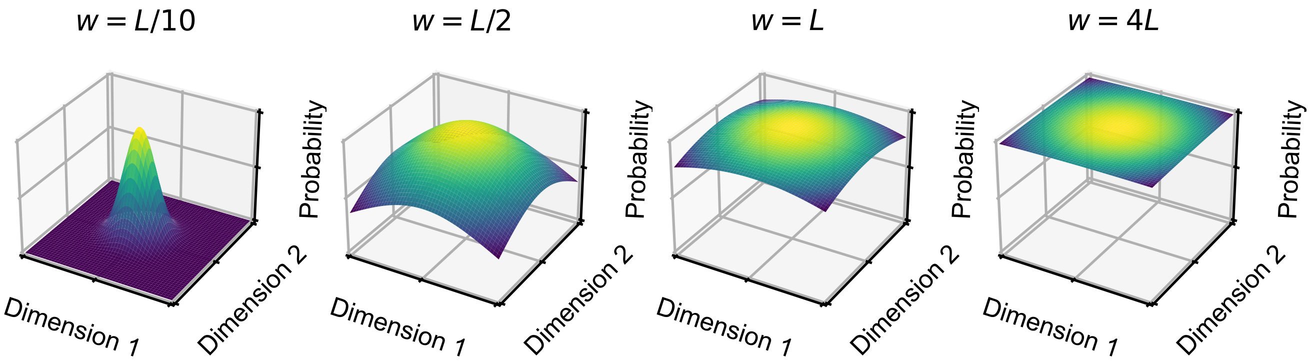

Fig. S1. Visualization of a Gaussian sampling kernel on a square habitat of size $L \times L$. Values of w are ranging from “narrow" to “broad".

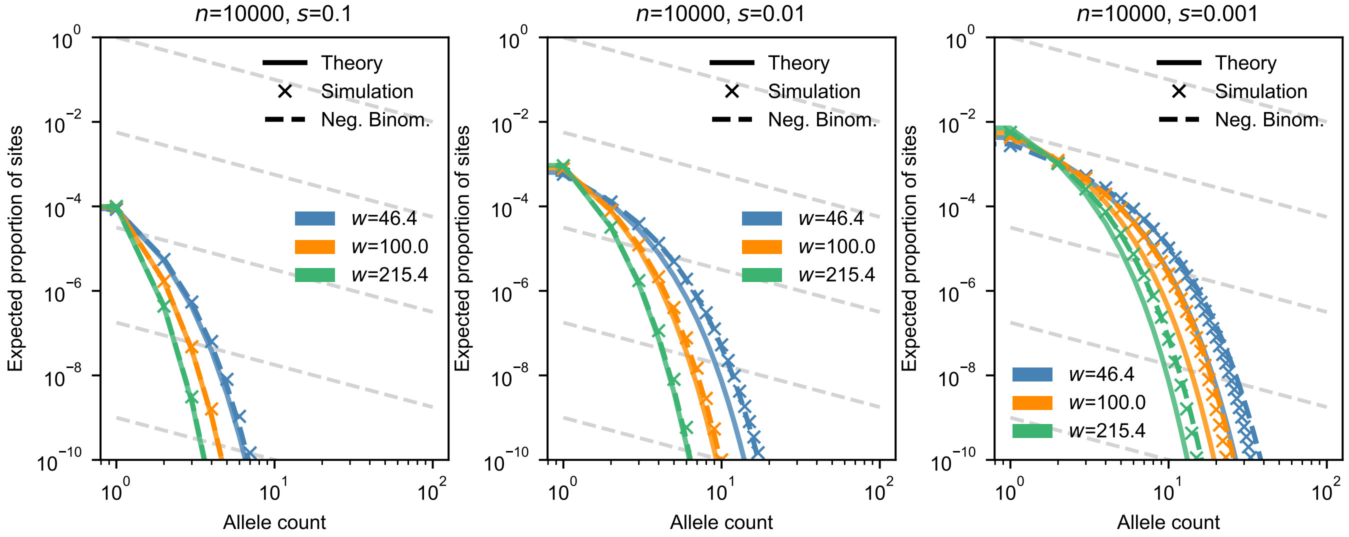

Fig. S2. Simulated frequency spectra (X's) with sample size $n = 10,000$ for a range of selection coefficients. Dashed lines indicate a negative binomial PMF fit to simulation results via the method of moments. Solid lines indicate theoretical expectation. Other model and simulation parameters include: $\sigma = 10$, $\rho = 20$, L=1,000, and $\mu = 10^{-9}$. Note that the negative binomal PMF is based on the underlying assumed Gamma distribution, and as such, the strong fit between theory and simulation here is taken as validation for the appropriateness of using a Gamma distribution to approximate the population allele frequency distribution.

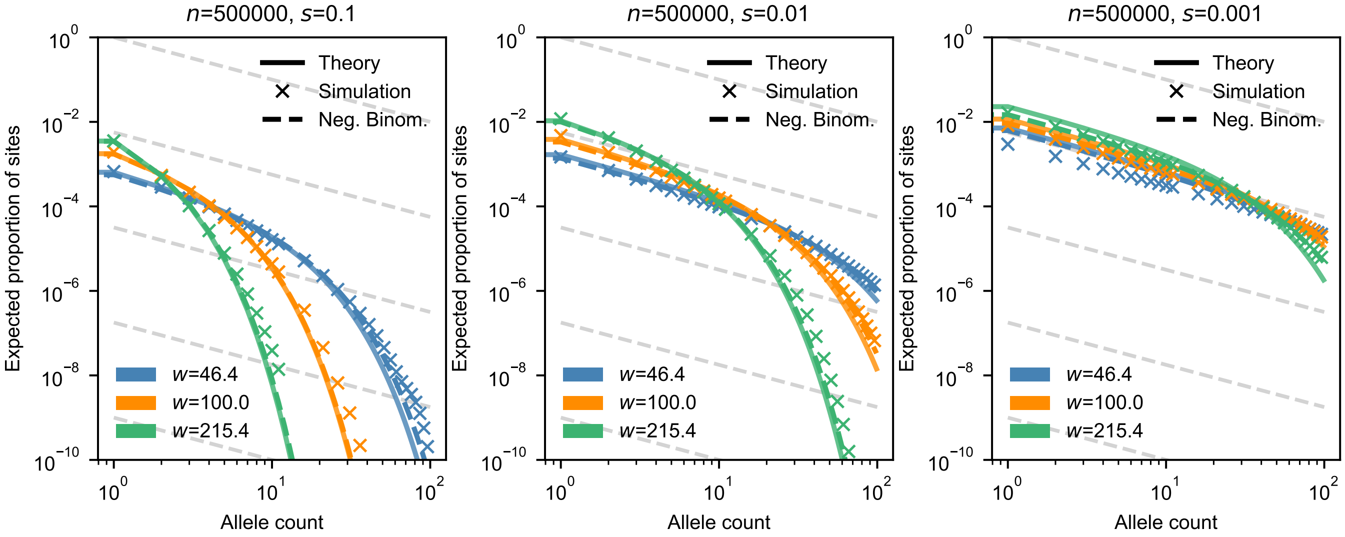

Fig. S3. Simulated frequency spectra (X's) with sample size $n = 500,000$ for a range of selection coefficients. Dashed lines indicate a negative binomial PMF fit to simulation results via the method of moments. Solid lines indicate theoretical expectation. Other model and simulation parameters include: $\sigma = 10$, $\rho = 20$, L=1,000, and $\mu = 10^{-9}$. Note that the negative binomal PMF is based on the underlying assumed Gamma distribution, and as such, the strong fit between theory and simulation here is taken as validation for the appropriateness of using a Gamma distribution to approximate the population allele frequency distribution.

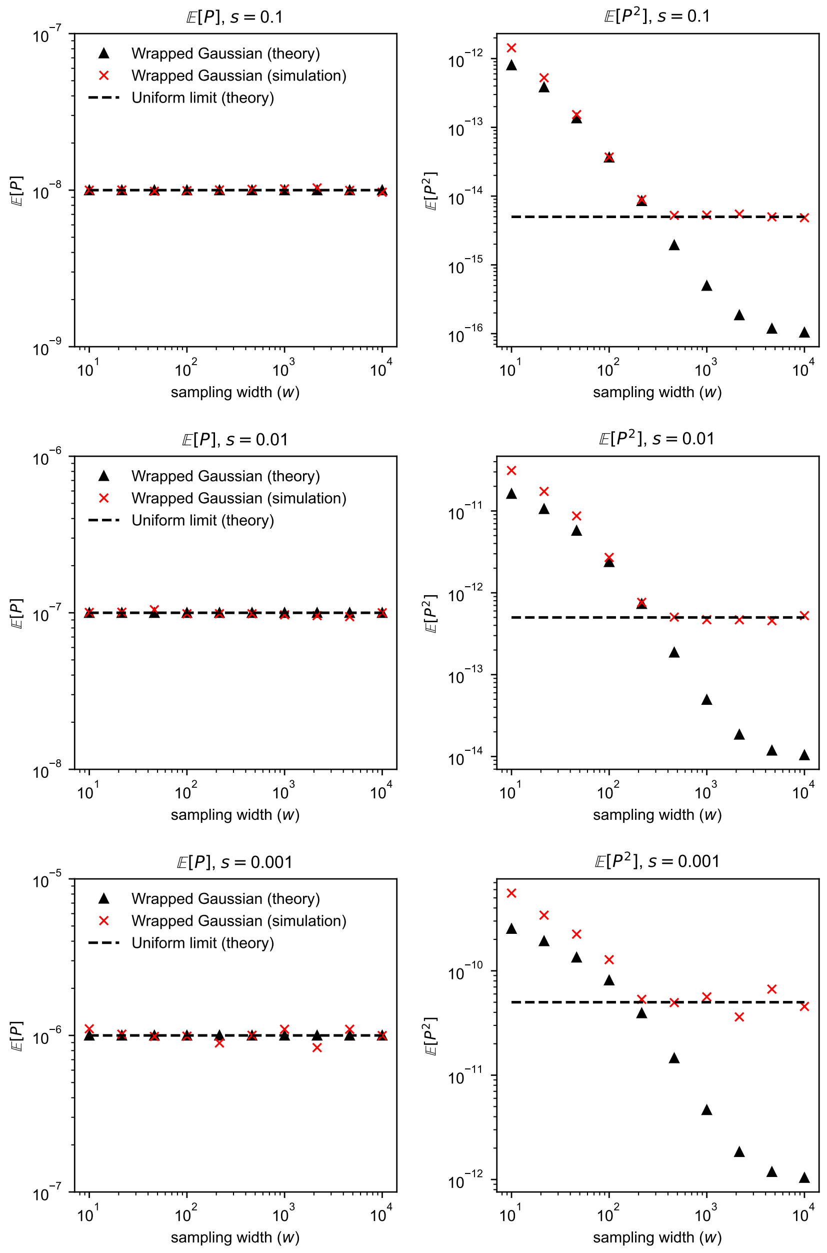

Fig. S4. Theory and simulation results for the first and second moments of P over a range of sampling widths and selection coefficients. Dashed line shows expectation under uniform sampling, while other markers indicate wrapped Gaussian sampling. Other model and simulation parameters include: $\sigma = 10$, $\rho = 20$, L=1,000, and $\mu = 10^{-9}$.

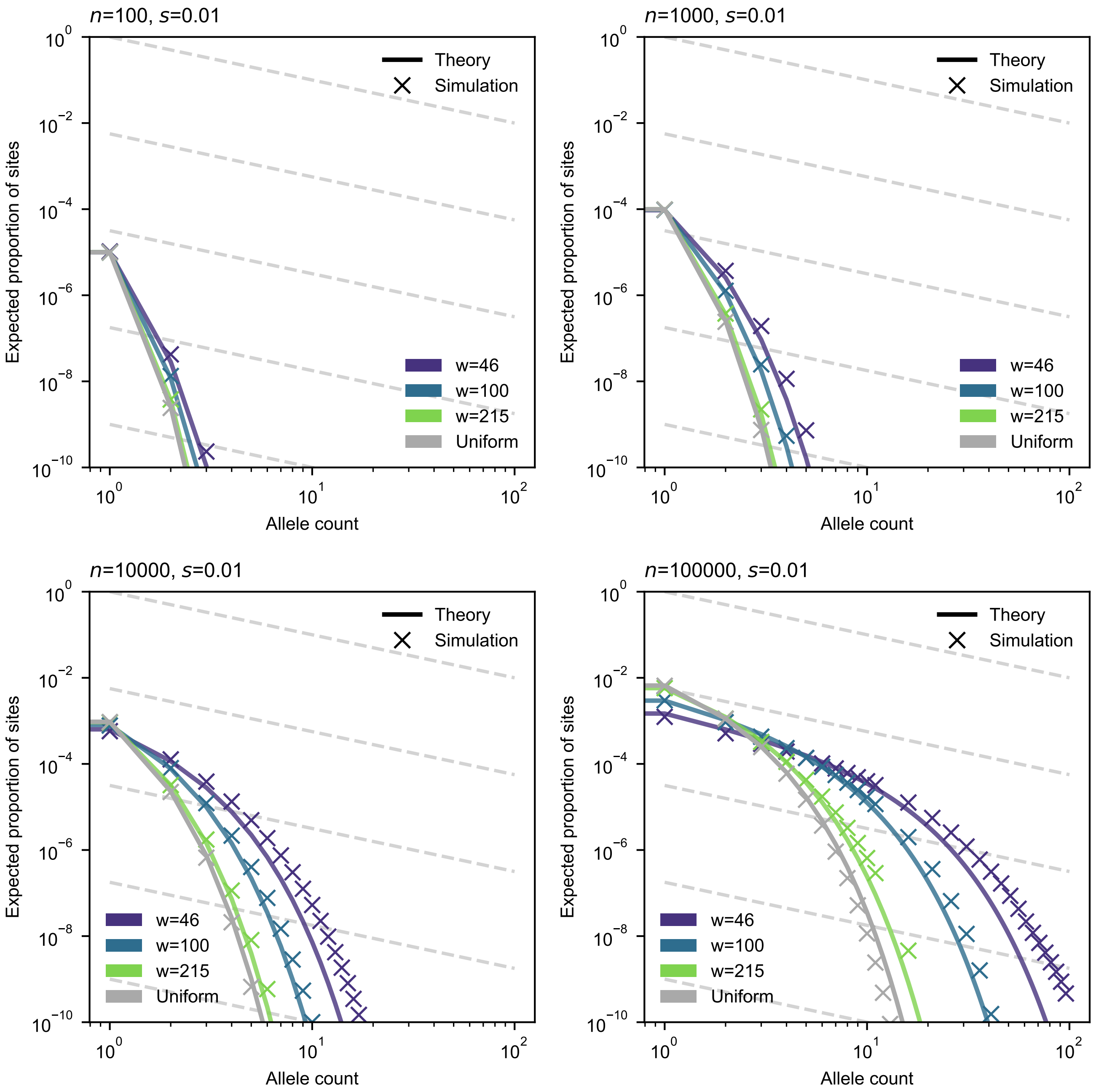

Fig. S5. Expected site frequency spectrum from theoretical model and branching process simulations for increasing sample size. Other model and simulation parameters include: $\sigma = 10$, $\rho = 20$, L=1,000, and $\mu = 10^{-9}$.

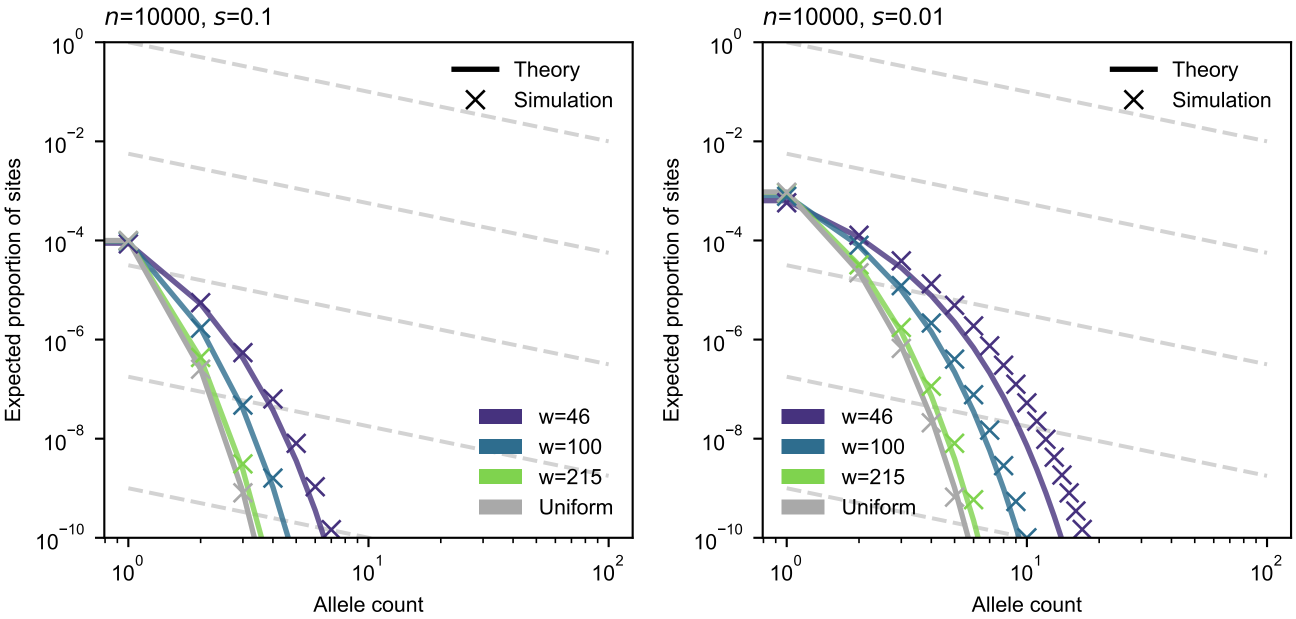

Fig. S6. Expected site frequency spectrum from theoretical model and branching process simulations for stronger (left) and weaker (right) selection, sample size $n = 10,000$. Other model and simulation parameters include: $\sigma = 10$, $\rho = 20$, L=1,000, and $\mu = 10^{-9}$.

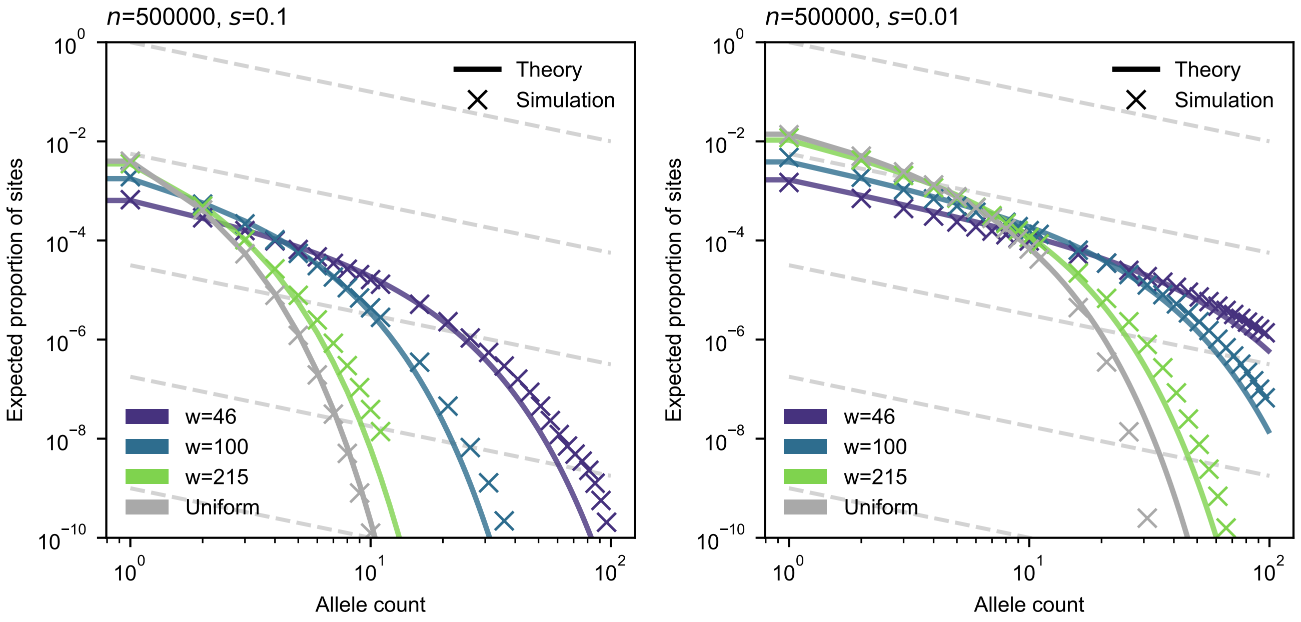

Fig. S7. Expected site frequency spectrum from theoretical model and branching process simulations for stronger (left) and weaker (right) selection, sample size $n = 500,000$. Other model and simulation parameters include: $\sigma = 10$, $\rho = 20$, L=1,000, and $\mu = 10^{-9}$.

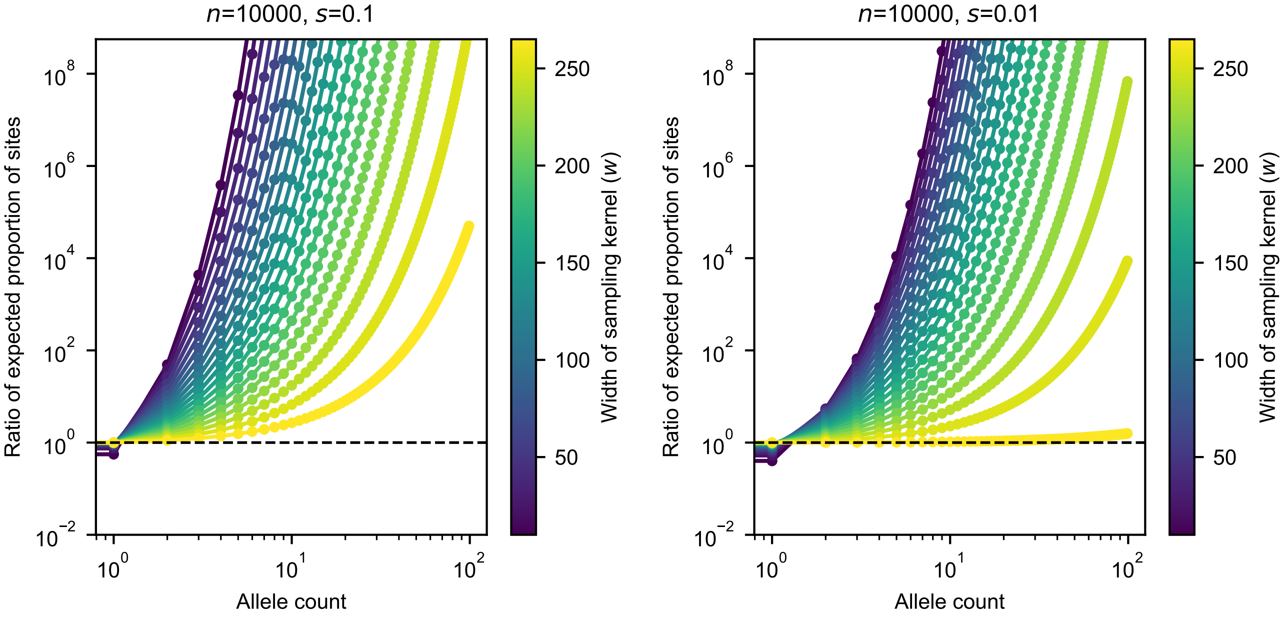

Fig. S8. Ratio between theoretical SFS values in a sample of width w vs. uniform sampling for stronger (left) and weaker (right) selection, sample size $n = 10,000$. Other model and simulation parameters include: $\sigma = 10$, $\rho = 20$, L=1,000, and $\mu = 10^{-9}$.

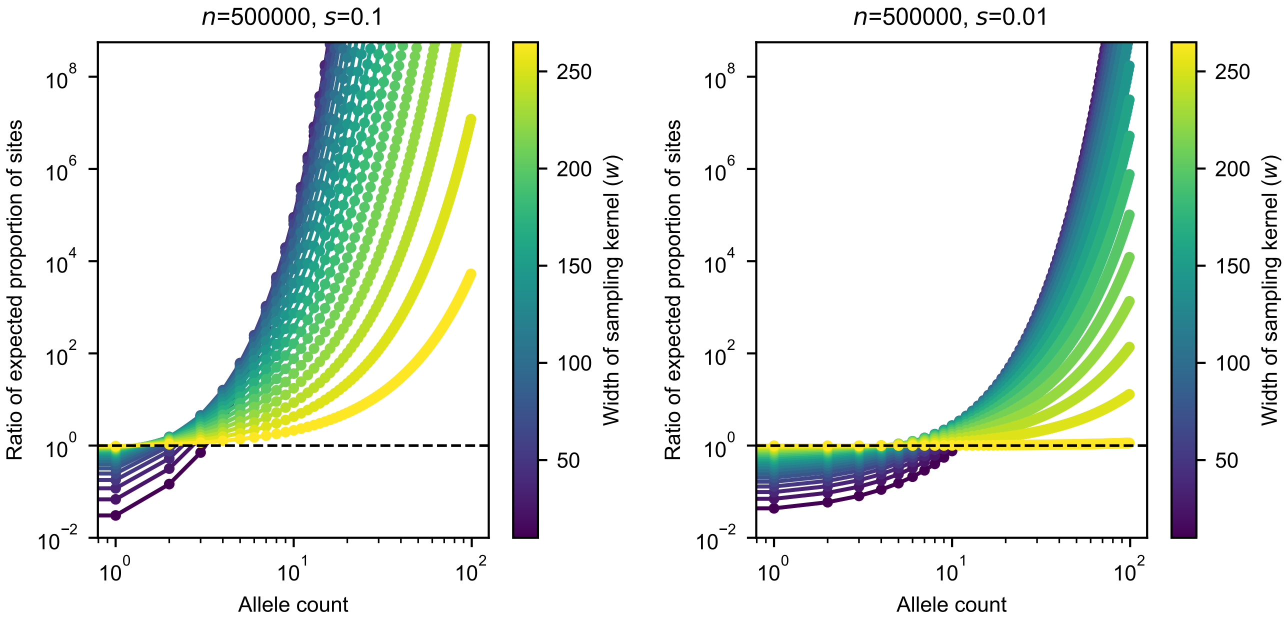

Fig. S9. Ratio between theoretical SFS values in a sample of width w vs. uniform sampling for stronger (left) and weaker (right) selection, sample size $n = 500,000$. Other model and simulation parameters include: $\sigma = 10$, $\rho = 20$, L=1,000, and $\mu = 10^{-9}$.

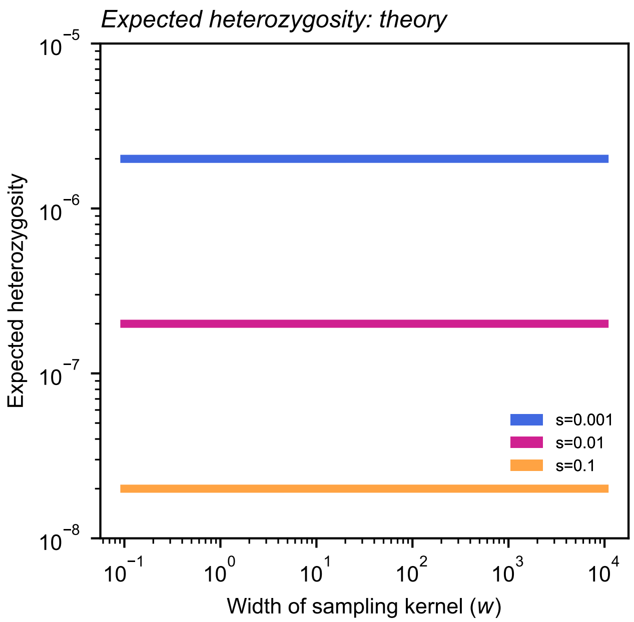

Fig. S10. Expected heterozygosity from theory. In plots shown, $\sigma = 10$, $\rho_N = 20$, and $\mu = 10^{-9}$.

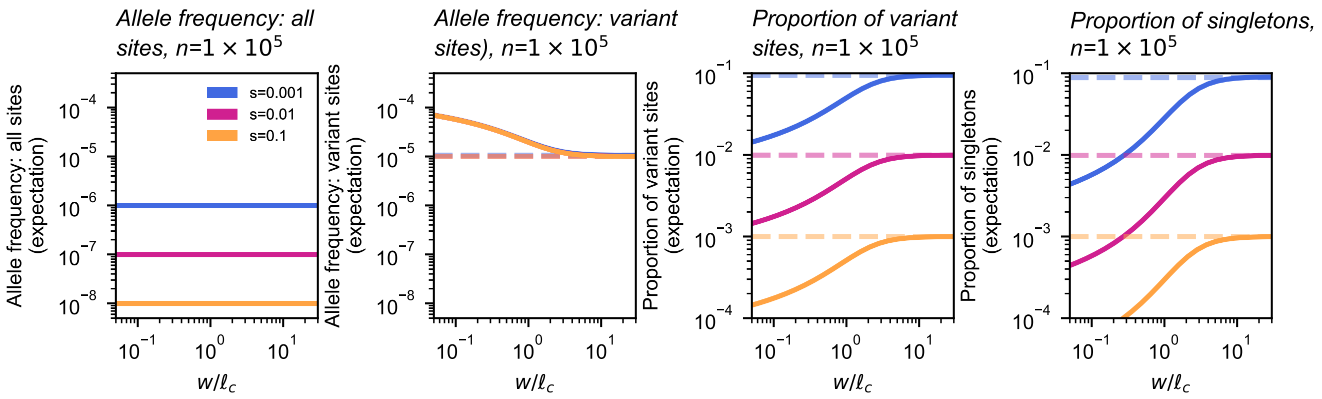

Fig. S11. Summary statistics, as in Fig. 4, as a function the scaled sampling width w/ℓ_c. Dashed lines represent theoretical expectation under uniform sampling. In plots shown, $\sigma = 10$, $\rho_N = 20$, and $\mu = 10^{-9}$.

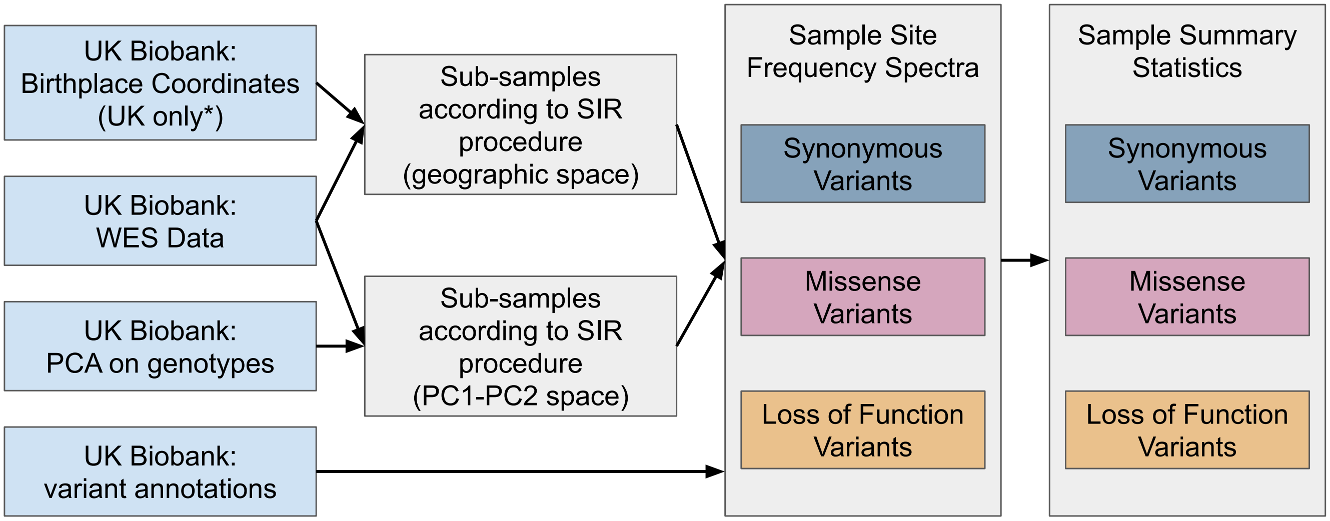

Fig. S12. Flowchart describing pipeline for data analysis in the UK Biobank. *Individuals were additionally filtered to those having high genetic similarity to the UKB centroid based on the first two principal components.

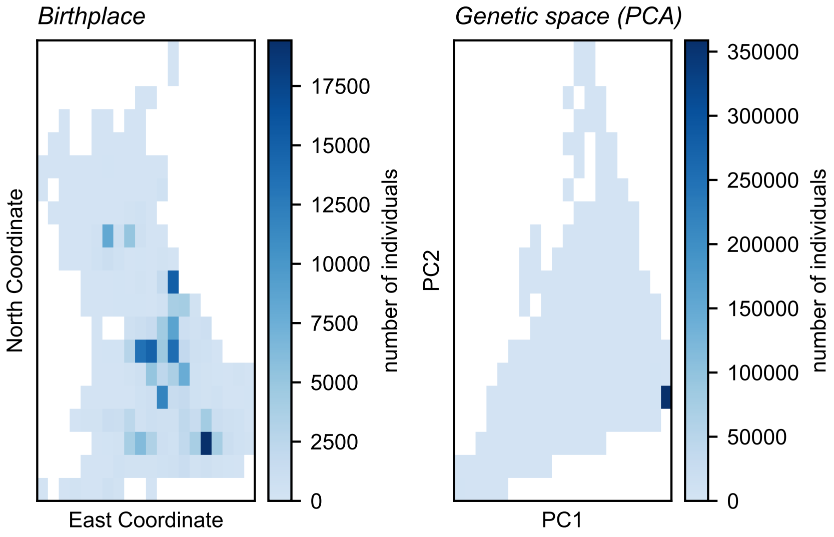

Fig. S13. Counts of individuals in the UK Biobank included in empirical analyses over discretized geographic (left) and genetic (right) space. Each grid has dimensions 20x20 with equal-sized bins.

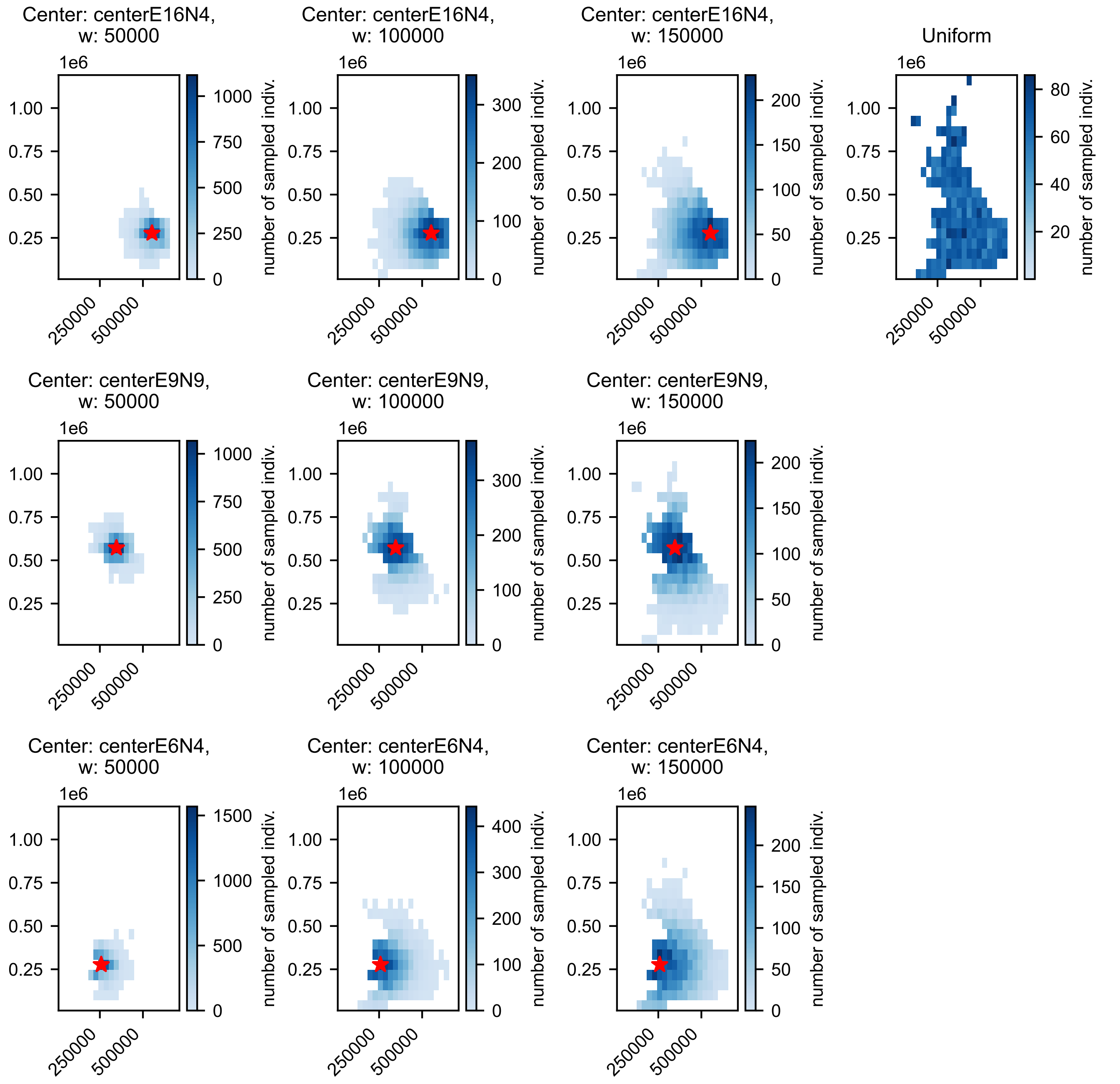

Fig. S14. Visualization of sampling distributions in geographic space across three center points and values of w (as well as the uniform distribution). Red stars indicate the sampling centers.

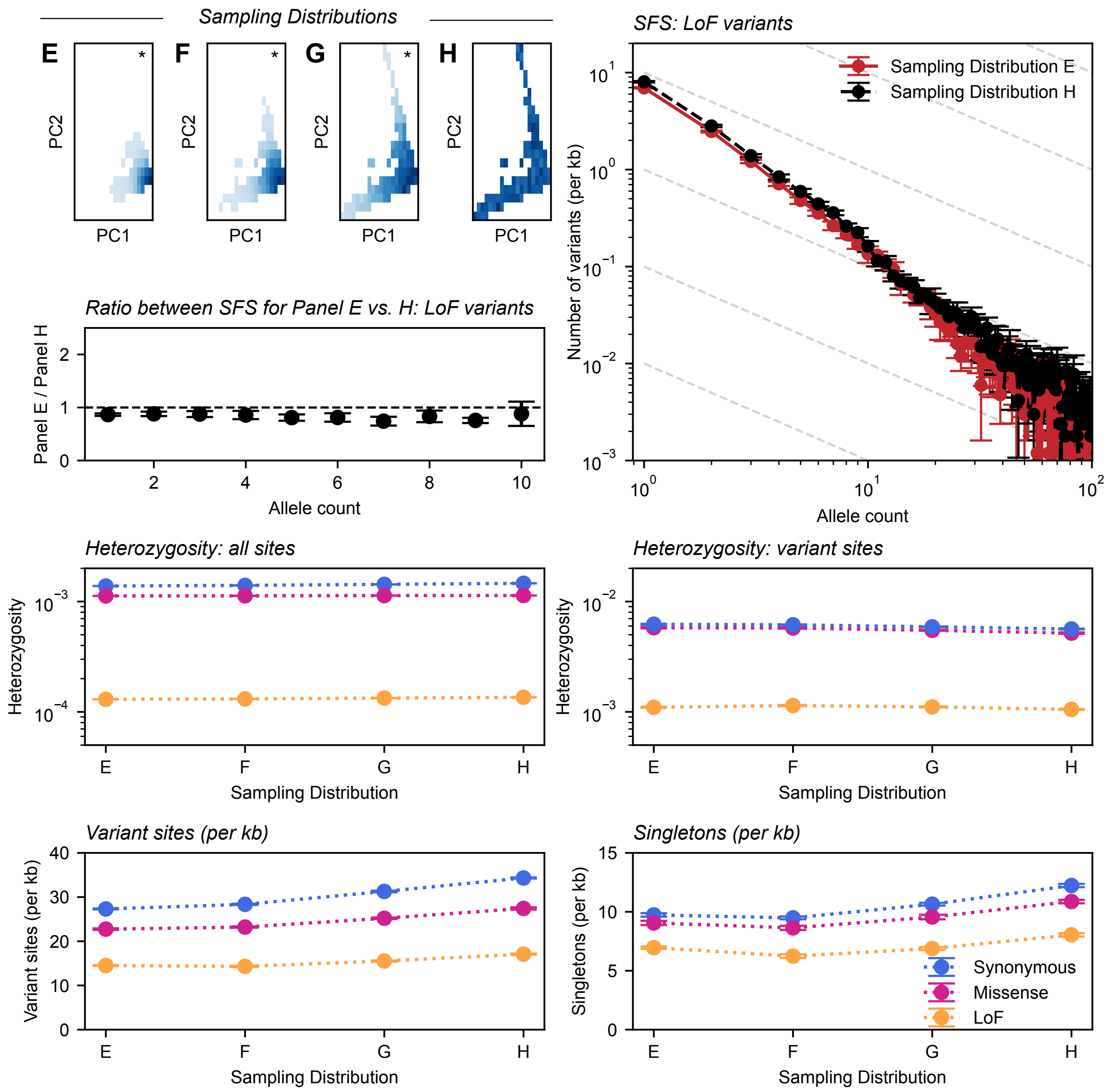

Fig. S15. Summary of results of PC-space sampling analysis in the UK Biobank. Panels are analogous to those of Fig. 6. Panels **E**-**H** depict Gaussian samples (of the entire dataset) over PC1-PC2 space centered at the median, with standard deviations 0.0015, 0,0025, and 0.005 units of Euclidean distance. Panel **H** depicts uniform sampling over PC1-PC2 space. SFS is truncated at allele count of 100. All results are averaged over ten sampling replicates with error bars representing two standard errors.

Heterozygosity (all sites)			
**Variant Type**	**E**	**H**	**Relative Change E→H**
**Synonymous**	1.383×10^{-3}	1.463×10^{-3}	5.78%
**Missense**	1.125×10^{-3}	1.134×10^{-3}	0.80%
**LoF**	1.300×10^{-4}	1.356×10^{-4}	4.31%
**Heterozygosity (variant sites)**			
**Variant Type**	**E**	**H**	**Relative Change E→H**
**Synonymous**	6.229×10^{-3}	5.640×10^{-3}	-9.46%
**Missense**	5.794×10^{-3}	5.174×10^{-3}	-10.70%
**LoF**	1.100×10^{-3}	1.053×10^{-3}	-4.27%
**Variant sites (per kb)**			
**Variant Type**	**E**	**H**	**Relative Change E→H**
**Synonymous**	27.31	34.32	25.67%
**Missense**	22.75	27.43	20.57%
**LoF**	14.85	17.08	15.02%
**Singletons (per kb)**			
**Variant Type**	**E**	**H**	**Relative Change E→H**
**Synonymous**	9.74	12.23	25.56%
**Missense**	9.05	10.87	20.11%
**LoF**	6.96	8.05	15.66%

Table S1. Relative change in summary statistics between narrow Gaussian and uniform sampling distributions in PCA-based re-sampling experiments across variant types. Sampling distributions are equivalent to those shown in Fig. S15. All results shown are averaged over ten sampling replicates.

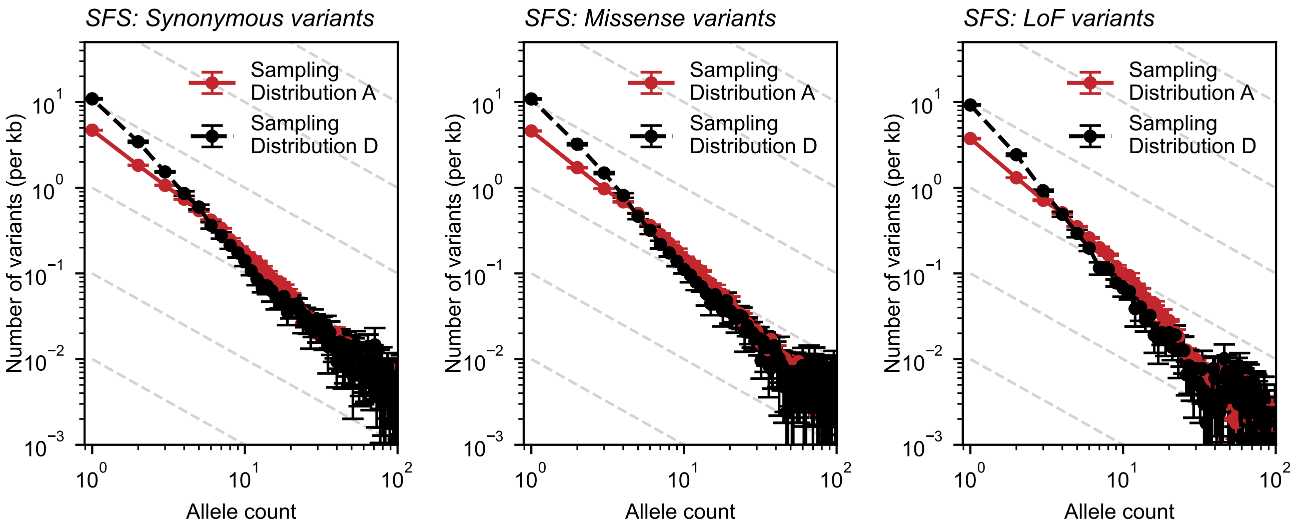

Fig. S16. Folded SFS for samples with distributions given in Fig. 6 panels A and D across variant types. Results for panel **A** are averaged across three sampling centers. All results shown are averaged over ten sampling replicates with error bars representing two standard errors. Synonymous and missense variants were randomly downsampled to the same number of SNVs as the LoF category; results shown per kb of exomic sequence.)

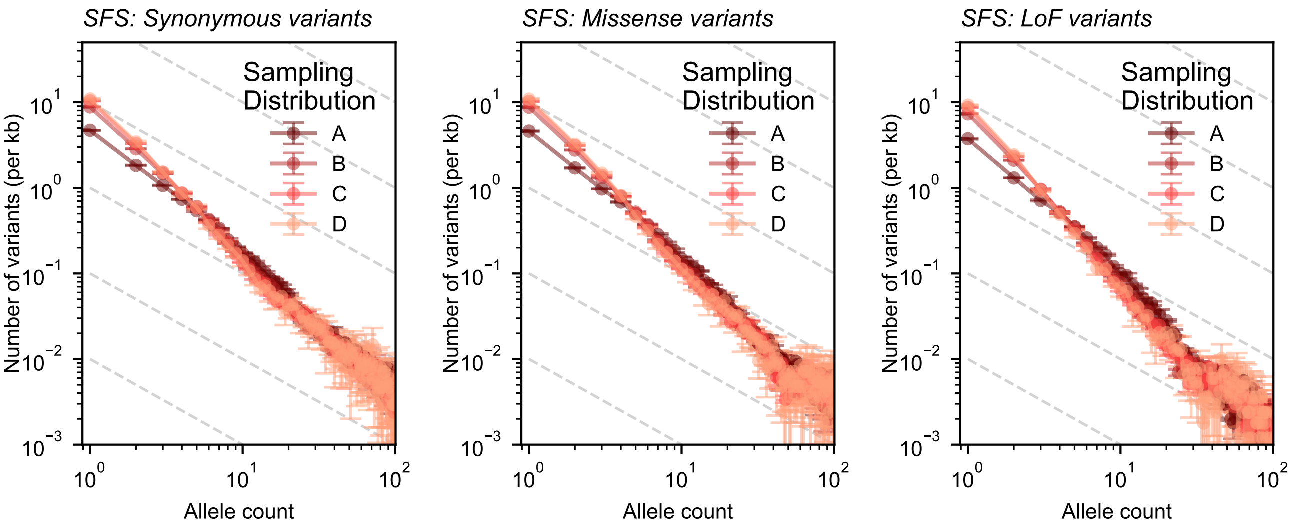

Fig. S17. Folded SFS for samples with distributions given in Fig. 6 across variant types. Results for panels **A**-**C** are averaged across three sampling centers. All results shown are averaged over ten sampling replicates with error bars representing two standard errors. Synonymous and missense variants were randomly downsampled to the same number of SNVs as the LoF category; results shown per kb of exomic sequence.

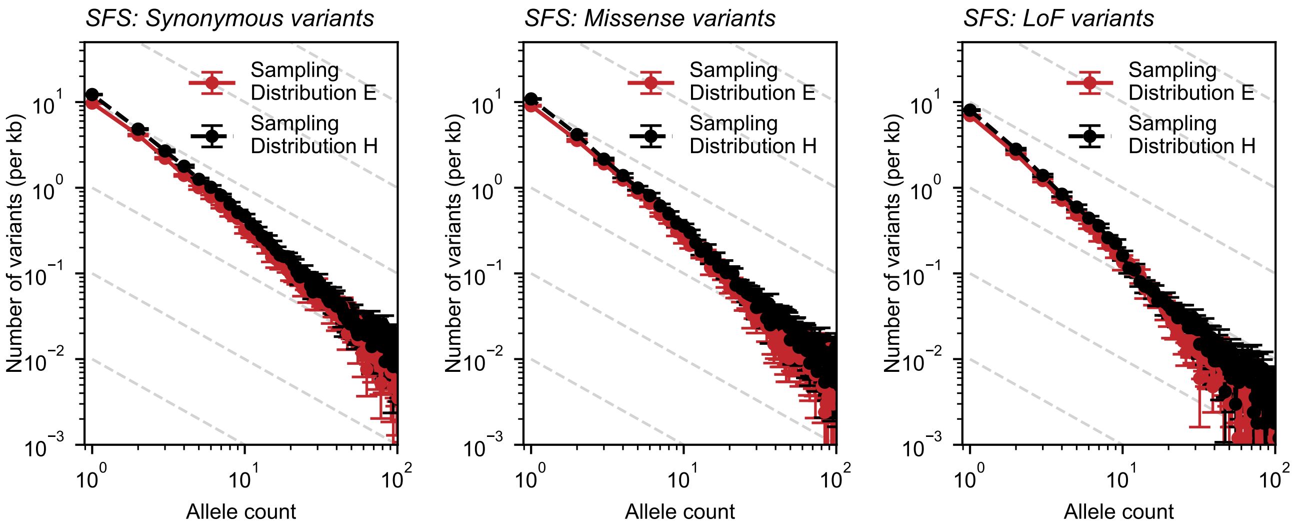

Fig. S18. Folded SFS for samples with distributions given in Fig. S15 panels **E** and **H** across variant types. All results shown are averaged over ten sampling replicates with error bars representing two standard errors. Synonymous and missense variants were randomly downsampled to the same number of SNVs as the LoF category; results shown per kb of exomic sequence.

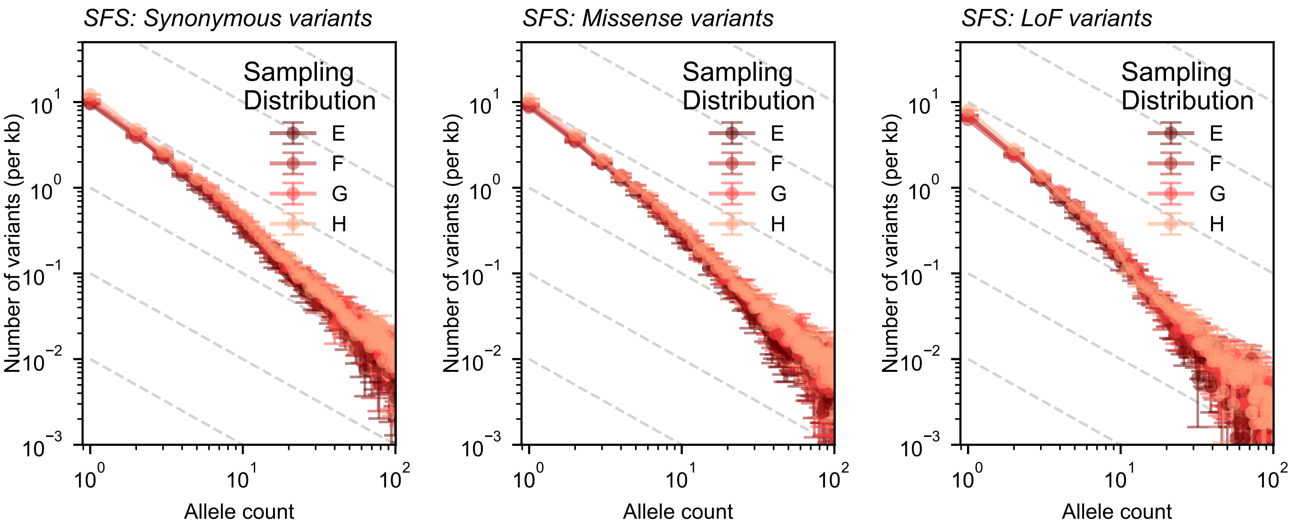

Fig. S19. Folded SFS for samples with distributions given in Fig. S15 across variant types. All results shown are averaged over ten sampling replicates with error bars representing two standard errors. Synonymous and missense variants were randomly downsampled to the same number of SNVs as the LoF category; results shown per kb of exomic sequence.

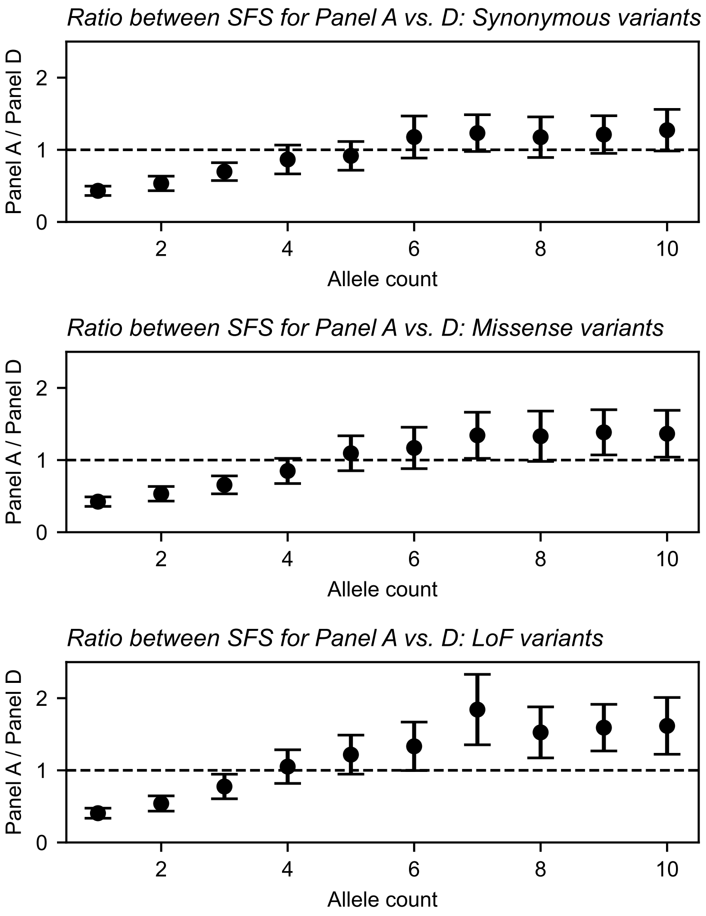

Fig. S20. Ratio between binned SFS values for panel **A** vs. panel **D** in Fig. 6. Results are averaged over ten sampling replicates and three sampling centers (Panel **A** only) with error bars representing two standard errors). Synonymous and missense variants were randomly downsampled to the same number of SNVs as the LoF category; results shown per kb of exomic sequence.

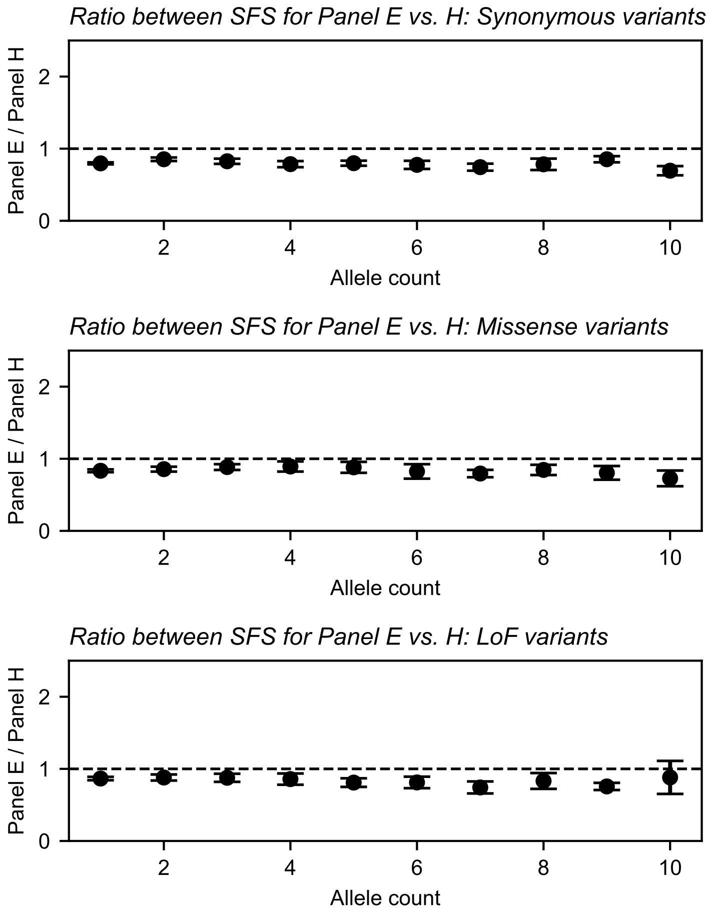

Fig. S21. Ratio between binned SFS values for panel **E** vs. panel **H** in Fig. S15. Results are averaged over ten sampling replicates with error bars representing two standard errors. Synonymous and missense variants were randomly downsampled to the same number of SNVs as the LoF category; results shown per kb of exomic sequence.

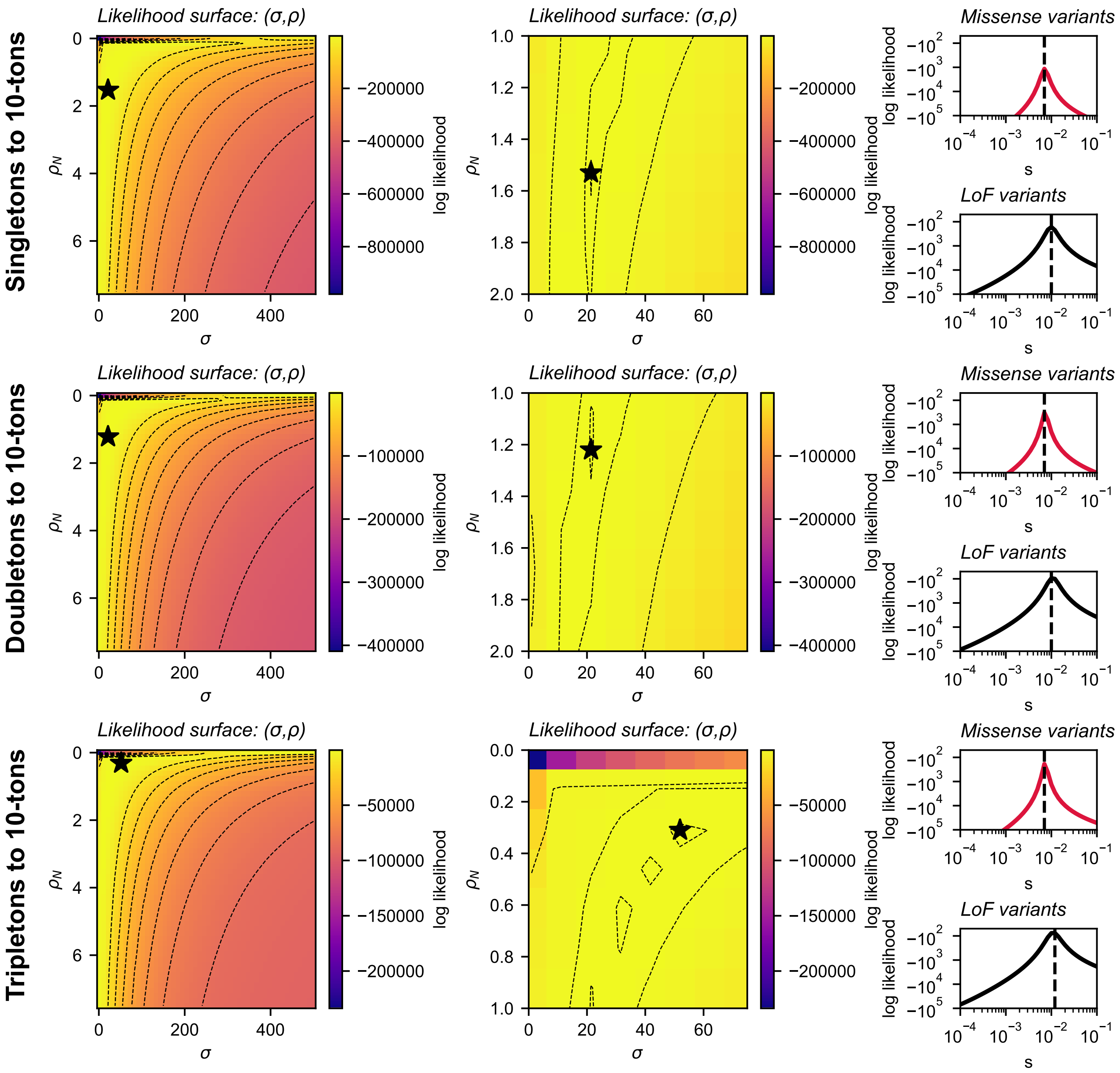

Fig. S22. (Left) Heatmaps with overlaid contour plots of log likelihood values for fit between theory and the SFS for missense variants. Log likelihood is computed as the sum of log likelihoods for samples with $w \in$[50km,100km,150km] (panels **A**-**C** in Fig. 6) given $\mu = 1.25 \times 10^{-8}$ and each combination of (σ, ρ_N, s) in the grid search. Counts from singletons to 10-tons (top), doubletons to 10-tons (middle), and tripletons to 10-tons (bottom) were used in the likelihood calculation. Values shown correspond to the values of s which maximize log likelihood given a particular pair of (σ, ρ_N). Star indicates the (σ,ρ_N) pair corresponding to the largest log likelihood (and, values used in Fig. S23). (Middle) Zoom-in of likelihood surface near maximum likelihood values. (Right) Likelihood surface of s for missense (top) and LoF (bottom) variants, given maximum likelihood values of (σ,ρ_N) fit to missense variants. Vertical dashed line indicates the value corresponding to highest log likelihood (used in Fig. S23).

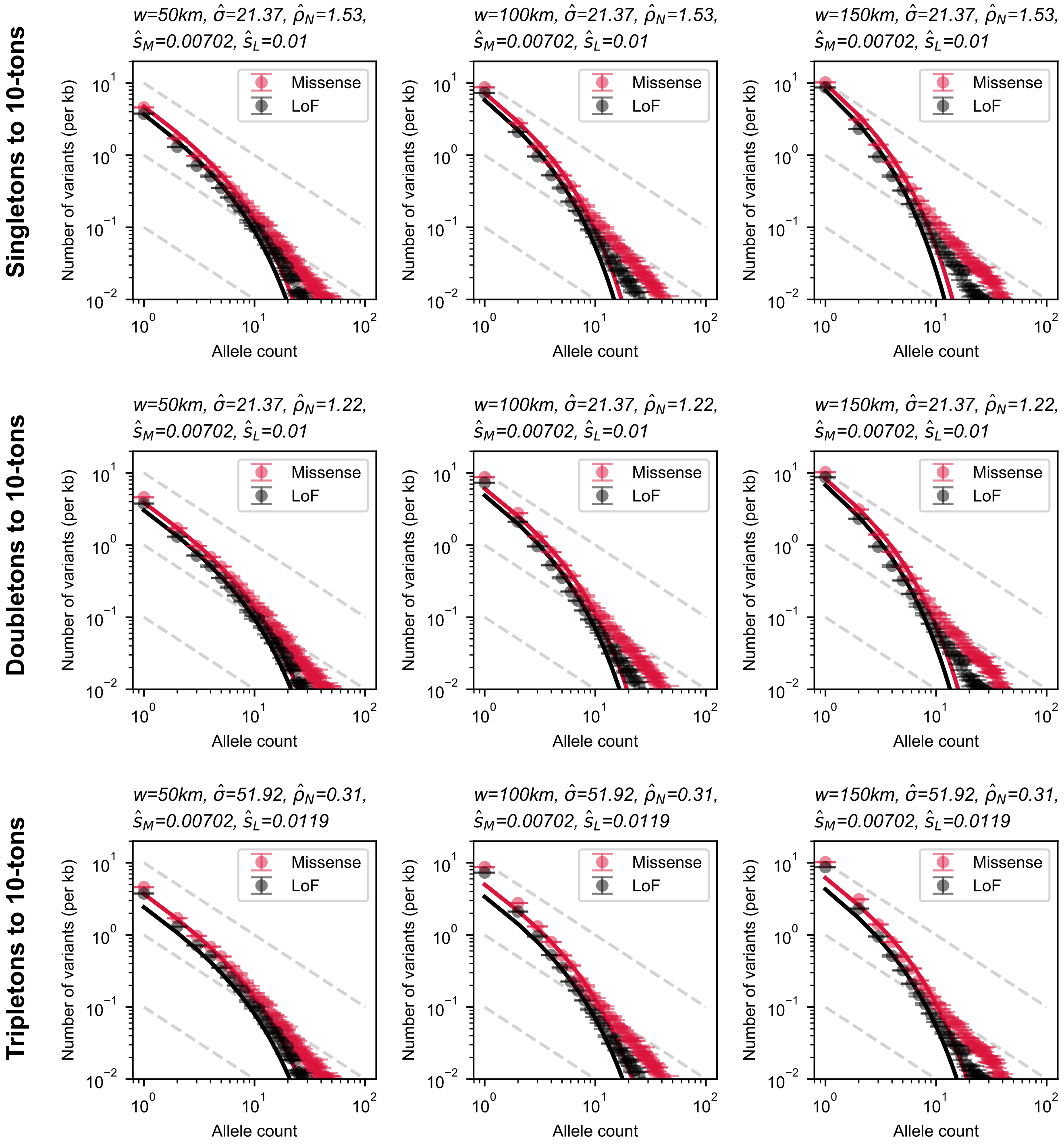

Fig. S23. Fitted frequency spectra for missense and LoF variants with extended allele count range (panels **A**-**C** in Fig. 6; increasing left to right) as compared to theoretical expectation using MLE parameter values ($\hat{\sigma}$, $\rho_{\hat{N}}$, and $\hat{s}_M$,Fig. S22; the value of $\hat{s}_L$ for LoF variants was chosen such that log likelihood of fit to the SFS of LoFs was maximized, given values of $\hat{\sigma}$ and $\rho_{\hat{N}}$ fit to missense variants). Results are shown varying the input data across counts from singletons to 10-tons (top), doubletons to 10-tons (middle), and tripletons to 10-tons (bottom).

References

1. M Friesen, Long-time behavior for subcritical measure-valued branching processes with immigration. *Potential Analysis* **59**, 705–730 (2023).
2. JBS Haldane, A mathematical theory of natural and artificial selection, part v: Selection and mutation. *Math. Proc. Camb. Philos. Soc.* **23**, 838–844 (1927).
3. J Novembre, M Slatkin, Likelihood-based inference in isolation-by-distance models using the spatial distribution of low-frequency alleles. *Evolution* **63**, 2914–2925 (2009).
4. ZB Hancock, RH Toczydlowski, GS Bradburd, A spatial approach to jointly estimate wright's neighborhood size and long-term effective population size. *Genetics* **227** (2024).
5. P Deraje, J Kitchens, G Coop, MM Osmond, The promise and challenge of spatial inference with the full ancestral recombination graph under brownian motion. *bioRxivorg* (2025).
6. S Wright, Isolation by distance under diverse systems of mating. *Genetics* **31**, 39–59 (1946).
7. S Watanabe, A limit theorem of branching processes and continuous state branching processes. *kjm.1* **8**, 141–167 (1968).
8. D Dawson, Measure-valued markov processes in *Ecole d'Eté de Probabilités de Saint-Flour XXI - 1991*, eds. DA Dawson, B Maisonneuve, J Spencer. (Springer Berlin Heidelberg), pp. 1–260 (1993).
9. JF Le Gall, *Spatial branching processes, random snakes and partial differential equations.* (Springer Science & Business Media), (1999).
10. A Etheridge, *An introduction to superprocesses.* (American Mathematical Soc.) No. 20, (2000).
11. K Kawazu, S Watanabe, Branching processes with immigration and related limit theorems. *Theory Probab. Appl.* **16**, 36–54 (1971).
12. G Malecot, Les mathematiques de l'heredite. (1948).
13. S Wright, Isolation by distance. *Genetics* **28**, 114–138 (1943).
14. GH Weiss, M Kimura, A mathematical analysis of the stepping stone model of genetic correlation. *J. Appl. Probab.* **2**, 129–149 (1965).
15. T Maruyama, Distribution of gene frequencies in a geographically structured population. 3. distribution of deleterious genes and genetic correlation between different localities. *Ann. Hum. Genet.* **36**, 99–108 (1972).
16. S Yokoyama, The rate of allelism of lethal genes in a geographically structured population. *Genetics* **93**, 245–262 (1979).
17. S Wright, Breeding structure of populations in relation to speciation. *Am. Nat.* **74**, 232–248 (1940).
18. M Kimura, T Ohta, Stepwise mutation model and distribution of allelic frequencies in a finite population. *Proc. Natl. Acad. Sci. U. S. A.* **75**, 2868–2872 (1978).
19. DT Gillespie, Exact stochastic simulation of coupled chemical reactions. *J. Phys. Chem.* **81**, 2340–2361 (1977).
20. CJ Battey, PL Ralph, AD Kern, Space is the place: Effects of continuous spatial structure on analysis of population genetic data. *Genetics* **215**, 193–214 (2020).
21. BC Haller, PW Messer, SLiM 3: Forward genetic simulations beyond the Wright–Fisher model. *Mol. Biol. Evol.* **36**, 632–637 (2019).
22. C Bycroft, et al., The UK biobank resource with deep phenotyping and genomic data. *Nature* **562**, 203–209 (2018).
23. T Zeng, JP Spence, H Mostafavi, JK Pritchard, Bayesian estimation of gene constraint from an evolutionary model with gene features. *Nat. Genet.* **56**, 1632–1643 (2024).
24. YB Simons, MC Turchin, JK Pritchard, G Sella, The deleterious mutation load is insensitive to recent population history. *Nat. Genet.* **46**, 220–224 (2014).
25. J Novembre, et al., Genes mirror geography within europe. *Nature* **456**, 98–101 (2008).
26. J Novembre, M Stephens, Interpreting principal component analyses of spatial population genetic variation. *Nat. Genet.* **40**, 646–649 (2008).
